# Molecular Mechanism of the Catalytic Radical Termination in Fatty Acid Photodecarboxylase

**DOI:** 10.1021/jacs.6c07143

**Published:** 2026-07-08

**Authors:** Giacomo Londi, Benedetta Mennucci

**Affiliations:** Department of Chemistry and Industrial Chemistry, University of Pisa, 56124 Pisa, Italy

## Abstract

Fatty acid photodecarboxylase (FAP) is a photoenzyme that converts fatty acids into hydrocarbons through light-driven radical chemistry. Although the early photochemical steps have been elucidated, the molecular mechanism governing the catalytic termination remains unresolved. Here, we address this issue through a multiscale computational investigation based on explicit dynamic simulations of the reactive processes through a combination of classical and polarizable QM/MM strategies. Our results indicate that, following rapid decarboxylation, the resulting alkyl radical is predominantly quenched via a proton-coupled electron transfer mechanism mediated by the protonated arginine R451 and nearby water molecules. In contrast, water-assisted bicarbonate formation is found to be a rare event at room temperature, consistent with recent experimental observations. Furthermore, calculated absorption spectra demonstrate that distinct active-site configurations, either involving a neutral R451/water network or the presence of bicarbonate, can both account for the transient red-shifted flavin species FAD_RS_ observed experimentally. Overall, these findings provide a coherent and unified molecular picture of the catalytic termination in FAP and emphasize the key roles played by active-site heterogeneity and water dynamics in controlling photoenzymatic reactivity.

## Graphical abstract

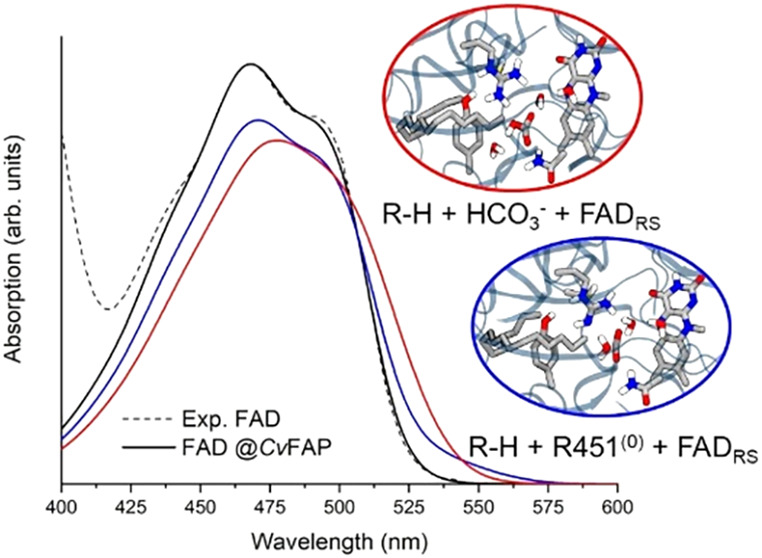

## Introduction

Photoenzymes represent an emerging frontier in biocatalysis, harnessing the energy of light to drive chemically challenging reactions under mild conditions.^1,2^ Unlike the vast majority of enzymes, which rely on thermal energy to overcome activation barriers, photoenzymes utilize an excited state to facilitate radical chemistry that would otherwise be thermodynamically difficult or impossible.^3–10^ In this context, fatty acid photodecarboxylases derived from the microalga *Chlorella variabilis* (*Cv*FAP) represent the most recently discovered class of naturally occurring photoenzymes.^11–13^ In particular, *Cv*FAP have attracted considerable attention for their ability to catalyze the light-driven oxidative decarboxylation of renewable free fatty acids (FAs) to produce C1-shortened *n*-alka­(e)­nes without requiring additional costly cofactors.^14,15^ This unique reactivity makes FAP a promising platform for sustainable fuel production and a model system for understanding radical-based enzymatic catalysis.

The *Cv*FAP catalytic cycle begins when the non-covalently bound oxidized flavin adenine dinucleotide (FAD) cofactor absorbs blue or violet light.^16^ In the singlet excited state ^1^FAD* becomes a much stronger oxidant, and can abstract an electron from the FA, present in the deprotonated form within the active site (R-COO^–^). The forward electron transfer (fET), observed to occur in ∼300 ps by means of transient absorption spectroscopy and time-resolved fluorescence measurements,^11,17^ leads to the formation of the anionic semiquinone FAD^•–^ and R-COO^•^ radical species, where the latter readily undergoes decarboxylation.

If, on the one hand, the first steps of the catalytic cycle were extensively investigated, both experimentally and computationally,^18–21^ on the other hand the termination steps still remain the subject of active debate. Different mechanisms were proposed for how the alkyl radical R^•^ is selectively quenched and converted into the final product R-H, while the oxidized FAD cofactor is restored. As shown in Figure 1, two main hypotheses are currently present in the literature. In the first one, Heyes et al. proposed that the cysteine C432 residue plays a crucial role:^22^ upon fast decarboxylation, a hydrogen atom transfer (HAT) would occur from C432 to R^•^, followed by a back (b)­ET from FAD^•–^ to the thiyl radical to form C432^(−)^ in ∼180 ns. Alternatively, Sorigué et al. proposed a pathway where the product R-H forms within 100 ns via the reduction of R^•^ by a bET from FAD^•–^, and such process is possibly coupled to a proton transfer (PT) mediated by a water molecule and the protonated arginine R451 residue. This hypothesis was supported by structural, computational, and spectroscopic results.^17^ Moreover, the conversion of a substantial fraction of CO_2_ into bicarbonate, concomitant with FAD^•–^ reoxidation, was further suggested. Recently, the mechanistic proposal, involving the bicarbonate anion HCO_3_^–^ as a plausible intermediate, has been revised by Bonvalet et al.^23^ Their new results, obtained by using time-resolved infrared spectroscopies, membrane-inlet mass spectrometry and isotope-exchange experiments, have confirmed the formation of HCO_3_^–^ at low temperatures (150–200 K), while excluding it at room temperature, where CO_2_ is observed to leave the active site in two different kinetic phases, in a few μs and in ∼ 400 μs. A not-yet-identified transient intermediate has been proposed to from, namely a CO_2_-derived species which does not involve any interaction with water molecules.

**Figure 1:**
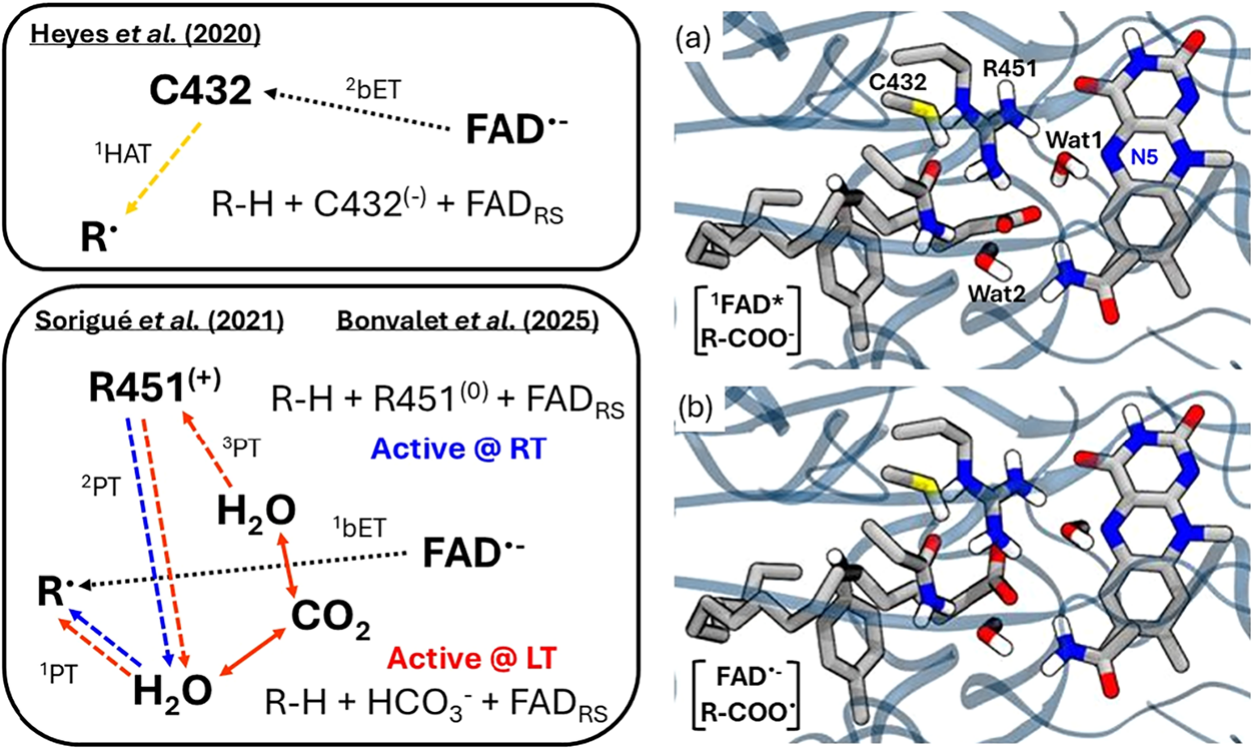
Proposed mechanisms for radical termination in *Cv*FAP: the one by Heyes et al. involving the participation of the C432 residue through a hydrogen atom transfer (HAT), followed by a back electron transfer (bET) from FAD^•–^ to the thiyl radical; the other by Sorigué et al. and, later on, by Bonvalet et al. suggesting two distinct pathways, which are active at different temperatures. At low temperatures (in red) the formation of the bicarbonate anion HCO_3_^–^ involves a single or a multi-proton transfer (PT) step: (1) from water to R^•^, coupled to the bET; (2) from the protonated R451 residue to a transient OH^–^ species; (3) from another water back to the deprotonated R451^(0)^. CO_2_ can interact with the transient OH^–^ generated by the first or the third PT. At room temperature (in blue), only the first two PT occur. On the right, the QM/MM optimized [^1^FAD* R-COO^–^] scenario (panel a) and the diradical [FAD^•–^ R-COO^•^] one (panel b).

Irrespective of the proposed termination mechanism, both studies observed the formation of a transient flavin species (FAD_RS_) which was shown to appear within hundreds of ns and as a result of bET from FAD^•–^. Such species was spectroscopically characterized through a red-shifted absorption spectrum compared to the oxidized FAD (see, for instance, Figure 2 in ref. 22 and Figure 5 in ref. 11). The origin of the FAD_RS_ species was initially attributed to the formation of the flavin thiolate charge-transfer species,^22^ and lately to a change in the electrostatic environment of the active site, likely due to the transformation of CO_2_ into the bicarbonate anion HCO_3_^–^, and/or to the formation of an H-bond between a water molecule (Wat1) and the N5 atom of FAD.^17^

**Figure 2:**
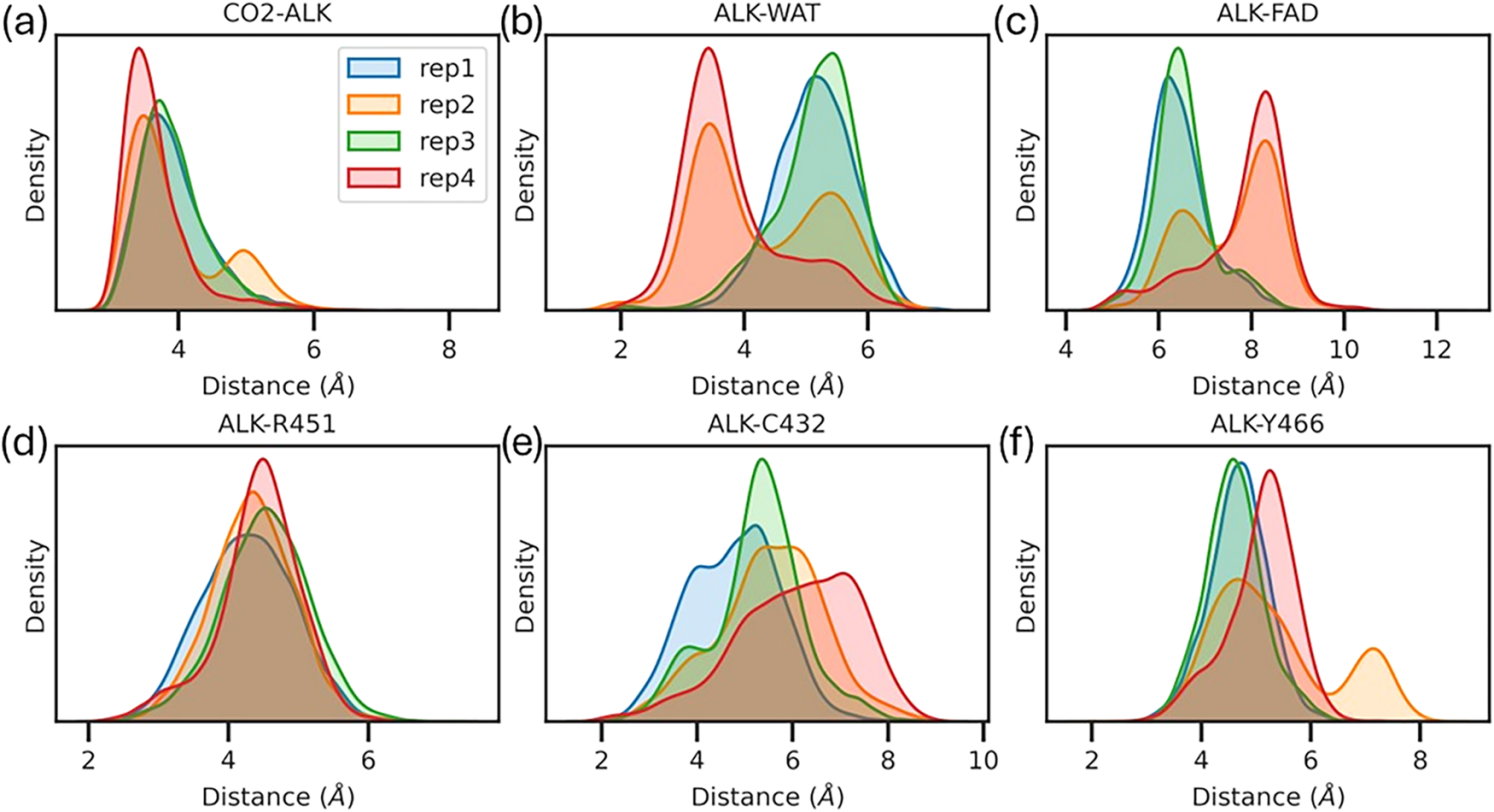
Distance distributions from the MD simulations of the four replicas of the [FAD^•–^ + R^•^ + CO_2_] system, reporting the minimum distance: between CO_2_ and the alkyl sp^2^ carbon-radical centered R’-H_2_C^•^ (panel a, see also Figure S7); between R’-H_2_C^•^ and the water hydrogens (panel b, see also Figure S9) or the N5 atom of FAD^•–^ (panel c, see also Figure S10) or the amino acid side chain heteroatoms’ hydrogens (panel d–f). Other distance distributions are shown in Figure S3.

In a previous work we performed a computational characterization of the structural and energetic factors of the photoactivation step in *Cv*FAP,^20^ by using a hybrid QM/MM strategy^24–30^ based on the polarizable AMOEBA force field.^31–35^ Through such an investigation we found that the interplay between protein-driven conformational and electrostatic effects is paramount in providing the correct energy matching (or redox potential), so that the excited ^1^FAD* can efficiently oxidize the FA substrate at the beginning of the photocatalytic cycle. According to our simulations, the fET process leading to the charge-transfer (CT) diradical [FAD^•–^ R-COO^•^] species involves the active participation of a water molecule (Wat1). In fact, as a result of the fET, Wat1 between the FA and the excited flavin rearranges to form an H-bond with the N5 atom of FAD, and at the same time the carboxylate group of the FA twists and shifts away from its original position, near the flavin core, as shown in Figure 1a,b. These structural rearrangements within the enzyme’s active site contribute to the CT state’s stabilization at the expense of the locally excited (LE) state on the flavin, thereby providing the driving force for the fET to occur within the experimentally measured time scales (∼300 ps). Furthermore, we also extended the polarizable QM/MM approach to static and dynamic simulations of the CO_2_ release in the active site. According to our calculations such process has a very low activation barrier of ∼2 kcal mol^–1^, suggesting that C–C bond cleavage occurs much faster (i.e., a few ps) than the fET step.

Building on these findings, in the present work we develop a multiscale computational framework (Section 1 of the Supporting Information) that combines room-temperature molecular dynamics (MD) simulations at both fully classical (MM) and polarizable QM/AMOEBA levels. By explicitly accounting for protein and solvent fluctuations, our aim is 2-fold: (i) to elucidate the termination mechanism leading to the formation of product R-H, and (ii) to unravel the origin of the transient FAD_RS_ species.

## Results and Discussion

In our previous work the analysis of the photoexcitation process was carried out on a different R451 rotamer with respect to the one observed in the crystallographic structure.^20^ As a preliminary step, we thus reinvestigated the photoexcitation process (fET and subsequent decarboxylation) on the crystallographic R451 rotamer, applying the protocol of ref 20. The semiclassical Marcus model (Section 2 of the Supporting Information and Figure S1) yields a fET time scale of ∼70 ps, in reasonable agreement with the experimental ∼300 ps and consistent with our earlier estimate on the alternative rotamer.^20^

Starting from the optimized CT diradical [FAD^•–^ R-COO^•^] structure (Figure 1b), two QM/AMOEBA MD replicas confirmed that decarboxylation occurs in <10 ps (Figure S2). Frames taken after this event then seeded extended MM MD trajectories of the [FAD^•–^ + R^•^ + CO_2_] system (four independent replicas of 250 ns). In these MM MD runs, we conveniently modified the MM force field by reparametrizing the atomic charges of the anionic semiquinone flavin and the alkyl chain to mimic the occurred fET and the presence of two radical species. Such simulations were performed with the purpose of allowing the newly formed CO_2_ and the alkyl radical R^•^ to sample the active site.

Analysis of the MD trajectories (Figure 2) indicates that the alkyl radical predominantly samples configurations compatible with a PT pathway involving R451 and nearby water molecules. The distance between R^•^ and FAD^•–^ is bimodal, with maxima at ∼6.5 and ∼8.5 Å (Figure 2c), reflecting two preferred poses of the radical. As R^•^ moves farther from the flavin, water molecules increasingly occupy the active site (Figure 2b), bringing at least one water molecule within ∼3 Å of the radical. At the same time, CO_2_ explores a broad configurational space during the MD simulations (Figure 2a and Figures S3–S4), indicating substantial active-site flexibility.

Consistent with a water-assisted PT mechanism, R^•^ remains relatively close to R451 (∼3–5 Å, Figure 2d), whereas broader and generally less favorable distance distributions are observed for C432 (∼4–9 Å, Figure 2e) and Y466 (∼4–6 Å, Figure 2f). These results support a PT from the guanidinium group of R451, potentially mediated by one or more water molecules, while suggesting a less prominent role for direct HAT from C432 or Y466. Notably, a recent computational study suggested that the thiol group of C432 may also act as a proton donor, leading to the formation of C432^(−)^.^36^ Previous investigations have shown that, although Y466 strongly interacts with the FA substrate during the early stages of the photocycle,^17^ it does not directly participate in either PT or HAT processes.^13,37–39^ Conversely, the possibility of a HAT pathway involving C432 cannot be ruled out *a priori*. However, in light of experimental evidence supporting a proton-coupled electron transfer (PCET) mechanism, the subsequent modeling presented here was restricted to this pathway leading to formation of the R-H product.

We selected 20 frames from each of the four replicas of the [FAD^•–^ + R^•^ + CO_2_] system within the 100–200 ns time-window as a starting point for QM/AMOEBA MD simulations (for a total of 80 trajectories), in which we assumed that the bET from FAD^•–^ to R^•^ has taken place, yielding the oxidized FAD and the R^–^ species, and yet still in the presence of CO_2_. In these simulations, the QM subsystem comprised the fragment R^–^, CO_2_, the side chain of R451, C432, and Y466 residues and all the water molecules within 5 Å from both the N5 atom of FAD, CO_2_, and the carbon atom bearing the negative charge in R^–^. The three selected residues were included as their side chains have p*K*_a_ *s* > 8 and their participation in the termination mechanism was suggested in earlier works.^11,17,22,40^

Among 80 QM/AMOEBA trajectories, 72 produced PT to the alkyl radical, thus showing that electron and proton transfer are intrinsically coupled. We note that 8 trajectories did not successfully reach any productive scenario within the explored simulations time window, and they were discarded from the subsequent analysis. Among the reactive trajectories, the PT to R^–^ follows four different scenarios (Figure 3):

(A) from a nearby water molecule which (as OH^–^) exchanges back and forth a proton with the protonated R451, and finally reacts with CO_2_, forming HCO_3_^–^ (1 trajectory, see Movie 1);
(B) from the protonated R451 either directly (27 trajectories, see Movie 2) or through a nearby water molecule (33 trajectories, see Movie 3). As a result, a deprotonated R451^(0)^ was found in ∼83% of the reactive cases;
(C) from the C432 thiol group, thus forming C432^(−)^ (7 trajectories);
(D) from the Y466 hydroxyl group, thus forming Y466^(−)^ (4 trajectories).

**Figure 3:**
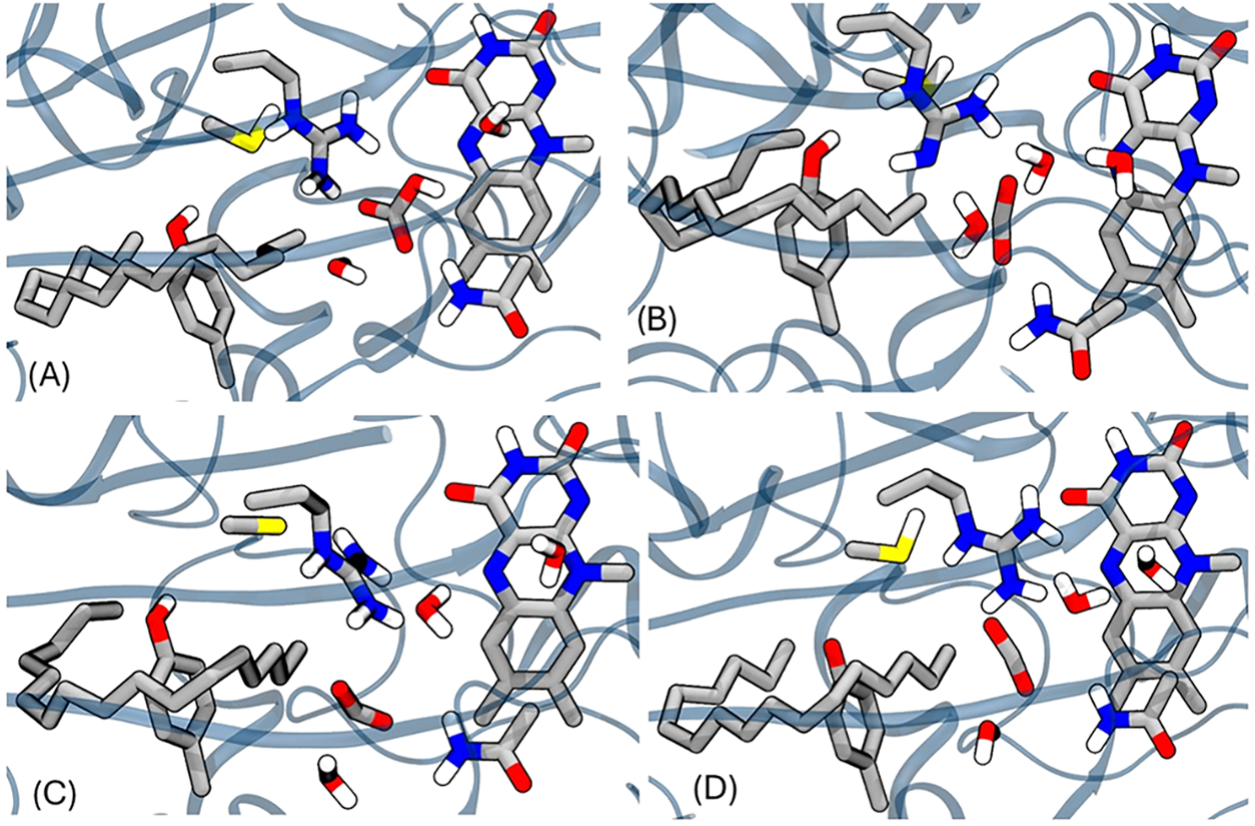
Representative snapshots of the four different scenarios obtained via QM/AMOEBA MD simulations, leading to the product R-H. Most of the trajectories involve a proton transfer from R451 either directly or mediated by water molecules (scenario B, ∼83%). Minor pathways include cysteine- (scenario C, ∼10%) and tyrosine-mediated proton transfer (scenario D, ∼ 6%), while bicarbonate formation is observed only once (scenario A, ∼ 1%). Other representative snapshots of scenario B are shown in Figures S13–S14.

In all cases, the PT process is completed in an ultrafast (sub-ps) process. Experimental evidence indicates that the combined bET+PT process is completed within approximately 100 ns. When considered together with our simulations, these findings suggest that the rate-determining step is the establishment of the conditions required for bET to occur.

To support the hypothesis that bET precedes and triggers proton transfer, we performed single-point QM/AMOEBA energy calculations on representative trajectories corresponding to the most probable Scenario B. Both the water-mediated PT pathway and the direct PT from R451 were considered. As shown in Figures S24–S25, the evolution of the total Mulliken charges on the flavin core and the alkyl chain clearly indicates that bET is the initiating event, driving the subsequent PT process, which takes place within a few tens of femtoseconds. In the water-mediated pathway, this time scale is sufficient for the water molecule initially hydrogen-bonded to the N5 atom of FAD^•–^ to reorient toward the newly formed negatively charged alkyl species and subsequently donate a proton. As a final analysis, we computed the electronic coupling between the two radical species for snapshots extracted from the [FAD^•–^ + R^•^ + CO_2_] replicas. Comparison of the resulting distributions (Figure S11) with the distance distributions shown in Figure 2c shows that not negligible coupling values are observed only when FAD^•–^ and R^•^ are at close distance.

Our QM/AMOEBA MD simulations indicate a main scenario (scenario B) where R451 plays a crucial role, thereby confirming its active participation in the termination mechanism as proposed by Sorigué et al.^17^ Interestingly, looking at the initial frames that produced this scenario (Figure S6), when the water molecule acts as the initial proton donor, the negatively charged carbon atom of R^–^ is generally closer (within ∼4 Å) to a water’s hydrogen than to a guanidinium group’s hydrogen of R451. Conversely, when the R451 acts as a direct proton donor, no water molecules were found in the vicinity of the alkyl fragment (>4 Å).

In our simulations, only one trajectory out of 80 yielded the bicarbonate anion HCO_3_^–^ formation through the combined participation of R451 and water (scenario A). This observation appears consistent with the findings of Bonvalet et al. from time-resolved IR measurements, which indicate that at room temperature, where CO_2_ can explore a larger configurational space in the active site, the formation of HCO_3_^–^ is less probable.^23^

To investigate how the transient R451^(0)^ can be rapidly back-protonated, 10 QM/AMOEBA trajectories giving scenario B were prolonged for further 10 ps. In these extensions we also increased the number of water molecules around R451^(0)^ treated at the QM level to achieve a more realistic picture of the possible reprotonation process. Within the selected simulation time, only in one trajectory we observed the back-protonation of R451^(0)^ by a water molecule, followed by the subsequent formation of HCO_3_^–^ by the transiently formed OH^–^ species (as in scenario A, Figure S15). In contrast, in the other trajectories a water molecule indeed remained in the vicinity of the deprotonated R451^(0)^, yet without back-donating a proton. In none of the trajectories we observed a PT from C432 to R451^(0)^, even though in some cases the distance between the C432 thiol group’s hydrogen and the guanidinium group’s nitrogen atoms of R451 was less than 3 Å (Figure S16). We note that the energy barrier for such a reaction calculated by Bonvalet et al. through QM/MM calculations was of the order of 10 kcal mol^–1^,^23^ which, according to the transition state theory, translates into kinetics of a few μs. Such time scales are indeed well beyond our QM/AMOEBA MD simulation times.

A key unresolved question concerns the origin of the transient FAD_RS_ species. To investigate such matter, we computed the absorption spectra for the dominant room-temperature pathway (scenario B), and for the initial dark-adapted state, that is before the photoexcitation (from now on, FAD @*Cv*FAP). To have a proper sampling of FAD @*Cv*FAP at room temperature, we performed three independent 1-μs long MM MD simulations of the solvated *Cv*FAP starting from the WT crystallographic structure. Some distributions of the minimum distances are reported in Figure S12. The strategy we adopted for the simulation of spectra is detailed in Section 1 of the Supporting Information. Here, we only recall that we performed TD-DFT/AMOEBA calculations of the FAD vertical excitation energies on ensembles of configurations extracted from either fully MM or QM/AMOEBA MD trajectories to include the static disorder and we added the vibronic features *a posteriori* via the spectral density formalism.^41–43^

The resulting absorption bands for FAD @*Cv*FAP and the main scenario B are reported in Figure 4a together with the experimentally measured spectrum of the WT *Cv*FAP in the dark.^11^ Considering first FAD@*Cv*FAP, the comparison with the experimental spectrum reveals that we accurately reproduce all the vibronic features. In terms of absolute peak positions, a blue-shift of ∼0.41 eV (62 nm) with respect to the experimental spectrum is observed. Such offset was used to consistently shift all the calculated spectra shown in Figure 4. The observed blue-shift originates from the underlying QM methodology, namely the use of TD-DFT and the specific choice of exchange-correlation functional.^44^ In this work, we used a state-of-the-art protocol for π-conjugated organic systems, based on an optimally tuned (OT) range-separated ωB97X-D scheme, and a comparison with CAM-B3LYP and B3LYP is provided in Table S1. Relative to the OT ωB97X-D results, CAM-B3LYP produces an additional blue-shift of approximately 80 meV, whereas B3LYP induces a red-shift of about 250 meV, thereby yielding excitation energies closer to the experimental values. Here, the systematic methodological error is not expected to affect the conclusions, as the analysis is based on relative spectral changes between different states rather than on the absolute positions of the absorption bands.

**Figure 4:**
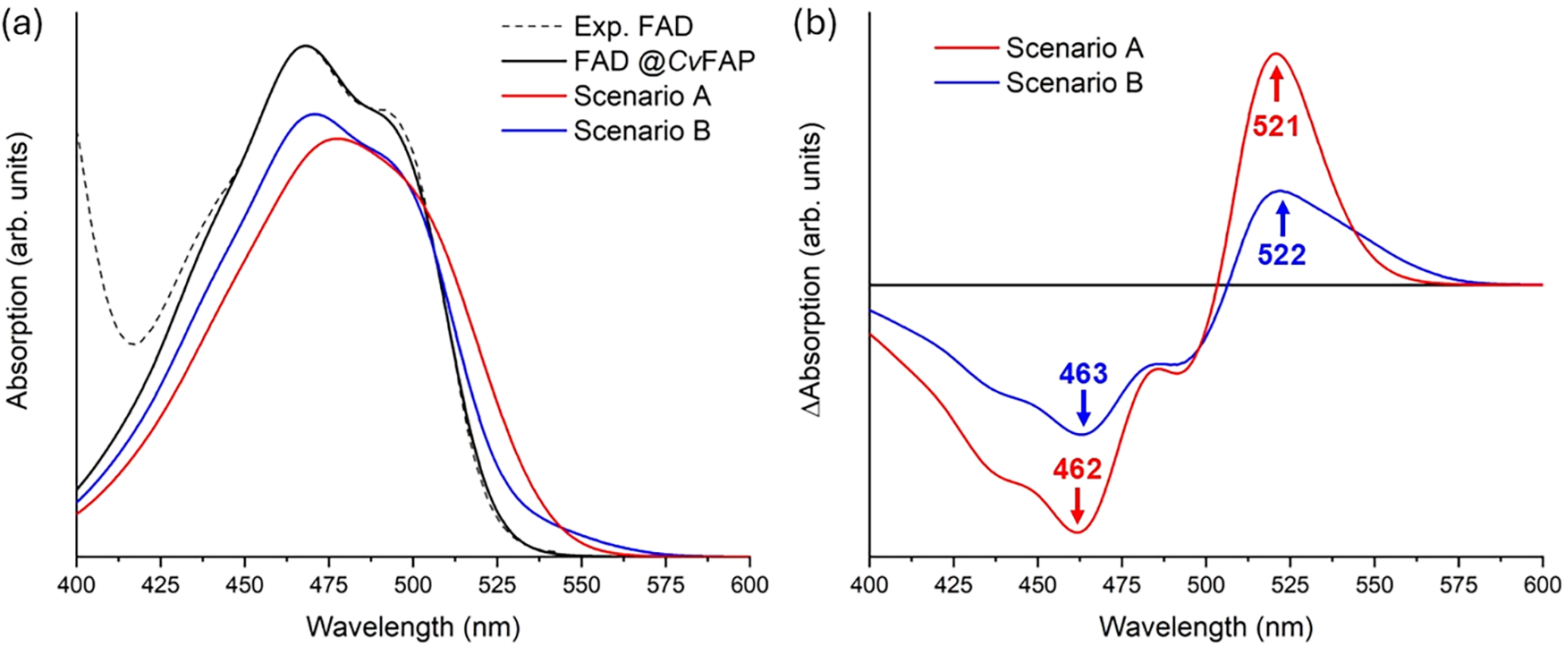
(a) Comparison between the normalized experimental absorption spectrum (black dotted line, digitalized from Figure 5B in ref 11.) and the calculated lowest-energy band of the FAD @*Cv*FAP (black solid line) and of scenario A (red) and B (blue). All the calculated spectra were shifted by 0.41 eV (62 nm); (b) difference of absorption spectra [scenario A - FAD @*Cv*FAP] and [scenario B - FAD @*Cv*FAP], where arrows point to the maxima of the FAD bleaching at around 465 nm and of the FAD_RS_ photoinduced absorption at around 520 nm.

Figure 4a shows that the calculated absorption spectrum of scenario B is slightly red-shifted and broader with respect to the initial FAD@*Cv*FAP. In the attempt to understand whether specific structural properties are responsible for such shift, we first investigated the internal structure of FAD in terms of its butterfly bending angle. As reported in Section 7 of the Supporting Information, in scenario B the bending observed in FAD @*Cv*FAP is maintained indicating that the observed variations in the excitation energies must arise from differences in the local environment. Then, we investigated the formation of an H-bond between a water molecule and the N5 atom of FAD, as this was suggested to be one of the possible origins of the FAD_RS_ species. In all the analyzed frames of scenario B, the formation of such H-bond occurred only in 6% of the analyzed structures. As shown in Figure 5a, where the FAD vertical excitation energies are plotted as a function of the minimum distance between the flavin and the neutral deprotonated R451^(0)^ species and the water, the maximum red-shift is indeed observed when R451^(0)^ is farther away from the flavin, and a water molecule can form an H-bond with the N5 atom of FAD. These results highlight the role of electrostatic fluctuations in tuning the flavin spectroscopic properties.

**Figure 5:**
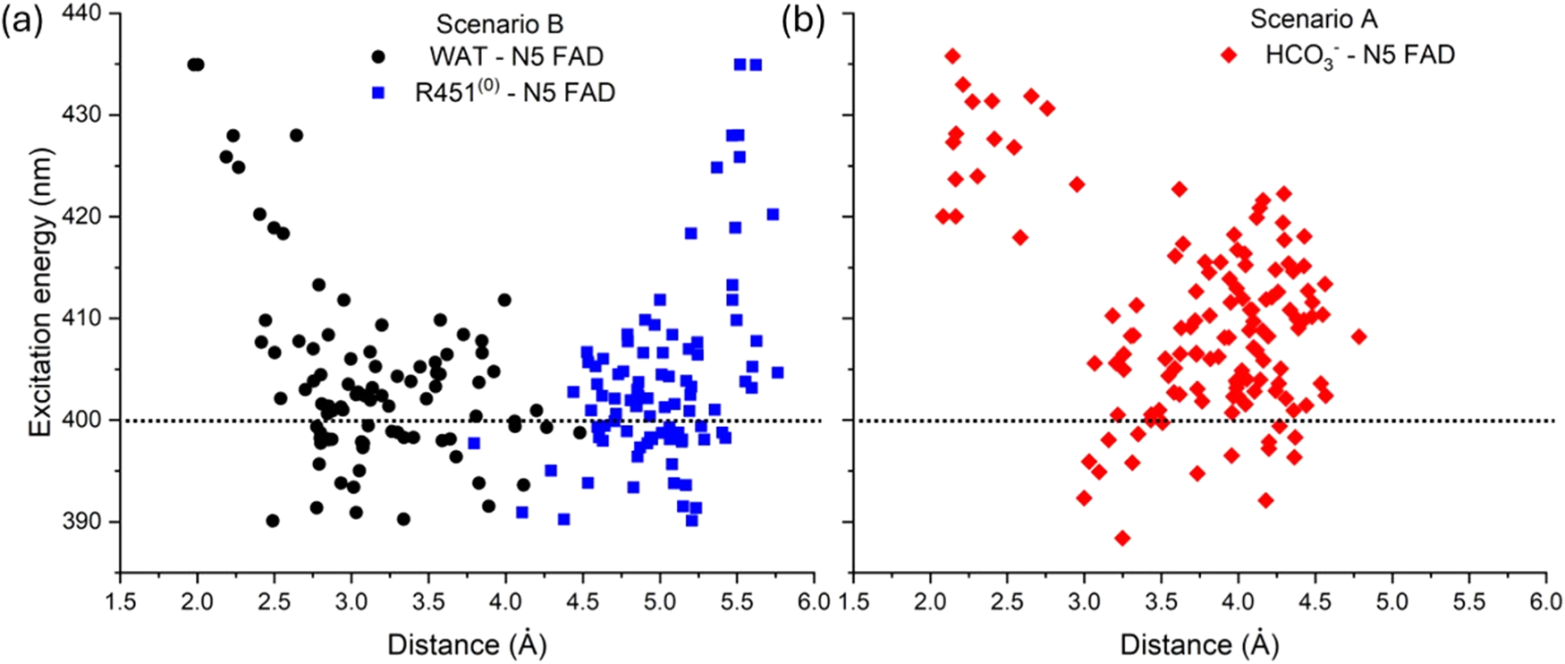
(a) The FAD vertical excitation energies as a function of the minimum distance between the N5 atom of FAD and the neutral deprotonated R451^(0)^ (in blue) and the closest water molecule (in black) in scenario B; (b) the FAD vertical excitation energy as a function of the distance between the N5 atom of FAD and HCO_3_^–^ in scenario A. The dotted black line at 400 nm marks the computed average FAD @*Cv*FAP excitation energy.

Another suggested possible origin of the observed transient FAD_RS_ species was the conversion of CO_2_ into HCO_3_^–^ within the active site. Because HCO_3_^–^ formation was observed only rarely in unbiased trajectories (once in scenario A, once among the extended scenario-B trajectories), additional biased QM/AMOEBA simulations were performed to generate a statistically meaningful ensemble for the simulation of the spectroscopic properties. Starting again from the trajectories giving scenario B, we ran new 10 ps long QM/AMOEBA MD simulations but this time limiting the QM region to R^–^, CO_2_, and the water molecules within 5 Å of the N5 atom, CO_2_, and the negatively charged carbon of R^–^, thereby preventing any competing PT from protein residues. Under these conditions all trajectories yielded HCO_3_^–^ within 2 ps (Figure S17). We emphasize that these simulations were intended solely to characterize the spectral signature of the bicarbonate scenario and not to estimate its probability. We then ran TD-DFT/AMOEBA calculations of the FAD vertical excitation energies on configurations extracted from the QM/AMOEBA trajectories using the same protocol used in the other scenario.

As shown in Figure 4a, the formation of HCO_3_^–^ (scenario A) red-shifts the absorption maximum with respect to the initial FAD@*Cv*FAP. Moreover, among the analyzed structures we also checked whether HCO_3_^–^ formed an H-bond with the N5 of FAD, as suggested by Farina et al.^45^ In < 10% of the cases we observed such interaction. However, in all cases in which HCO_3_^–^ was found within 3 Å from the flavin core, the FAD excitation was red-shifted by about 30 nm relative to FAD@*Cv*FAP (Figure 5b). As expected, the presence of a negative charge close to FAD has the effect of red-shifting its excitation energy,^46^ and even more so when HCO_3_^–^ interacts through an H-bond, for which an explicit orbital mixing with FAD was found (Figure S22). To quantify the effect of this mixing, we repeated the calculations by treating only the flavin core at the QM level, while all the rest with the polarizable AMOEBA description. In this way we removed possible quantum effects on the FAD excitation due to the presence of other QM fragments, and particularly HCO_3_^–^. The correlation analysis of the FAD excitation calculated with the two different QM regions (Figure S21) clearly shows that all the configurations that deviate from linearity indeed have the bicarbonate anion within 3 Å to FAD. Thus, both electrostatic interactions and orbital mixing contribute to the observed FAD spectral changes.

As a final analysis of the origin of FAD_RS_, Figure 4b shows the difference absorption spectra obtained by subtracting the FAD@*Cv*FAP spectrum from those of scenario B and A. Both scenarios give a very similar picture, well reproducing the experimental measurements reported by Heyes et al., namely a positive peak at around 520 nm (517 nm experimentally) and a negative peak at around 465 nm (465 nm experimentally). However, the magnitude of the red-shift of the band maxima is different in the two scenarios (Figure 4a): 10 nm for scenario A vs 4 nm for scenario B. This shows that the features of the difference absorption spectra depend not only on the energy shift but also on the change in the band shape. In fact, our calculations for the two scenarios show a larger broadening than for the initial FAD@*Cv*FAP, consistent with experimental observations.^19^ Since the experimental spectra were recorded at cryogenic temperatures,^19^ we additionally computed absorption spectra at 77 and 150 K (Figure S23). Under these conditions, the spectra of FAD@*Cv*FAP and scenario B accurately reproduce the experimental spectra measured before and after illumination, respectively, whereas scenario A tends to overestimate the observed spectral changes.

Finally, we recall that Bonvalet et al. recently observed that the bicarbonate anion HCO_3_^–^ forms only at low temperatures (150–200 K),^23^ and yet the FAD_RS_ is found across all temperatures. Our calculations reconcile this apparent inconsistency, showing that the transient FAD_RS_ can arise from distinct structural arrangements, including either HCO_3_^–^ or R451^(0)^/water located in close proximity to FAD.

More generally, the results suggest that the temperature-dependent mobility of CO_2_ and water molecules plays a key role in determining the termination pathways. At room temperature, CO_2_ is expected to more easily diffuse away from R^•^, which in turn can shift from its initial position, thereby allowing water molecules to occupy the active site. Under such circumstances, we may reasonably expect that the ultrafast PT (coupled to the bET) is likely to involve a water molecule. The resulting transient OH^–^ can then quickly abstract a proton from R451 and interact with FAD, leading to FAD_RS_ formation. Conversely, at low temperatures, the mobility of CO_2_ is reduced and, when the transient OH^–^ is formed as a result of the PT, it is more likely to react with the nearby CO_2_, leading to HCO_3_^–^ and an alternative structural origin of the same spectroscopic signature.

## Conclusions

In summary, our multiscale computational investigation, combining explicit dynamic simulations with classical and polarizable QM/MM approaches, provides a mechanistic picture of radical termination in the WT *Cv*FAP catalytic cycle. Our simulations indicate that electron and proton transfer are intrinsically coupled processes, thereby ensuring efficient quenching of the reactive alkyl radical. In particular, at room temperature, the dominant PCET mechanism involves the conserved R451 residue acting in concert with dynamically organized water molecules, whereas bicarbonate anion formation appears to represent a less probable pathway. We acknowledge that the statistical sampling of the proton-transfer process remains limited; however, the pathways identified in our simulations are consistent with the available experimental evidence. Therefore, rather than definitively assigning a unique operative mechanism in *Cv*FAP, our results provide a probabilistic description of the most likely reactive pathways governing radical termination.

This work also highlights the critical role of active-site heterogeneity and water dynamics throughout the whole catalytic cycle of *Cv*FAP, thereby tuning both reactivity (from favoring the initial fET to contributing to the formation of the final product R-H) and the spectral properties of FAD. Indeed, our results show that the experimentally observed FAD_RS_ does not reflect a single, well-defined environment. Rather, significantly different configurations, such as the presence of the bicarbonate anion (a scenario which could be active at low temperatures) or the scenario found at room temperature where R451 has donated a proton, yielding a neutral residue, and water molecules can interact with FAD, can explain the spectroscopic features observed in the formation of FAD_RS_. This finding reconciles previously reported experimental observations and stresses the importance of considering ensemble effects when interpreting spectroscopic data in enzymatic systems.

Finally, our results emphasize that water-mediated PCET serves as a powerful natural blueprint for stabilizing and directing highly reactive radical intermediates. The broader impact of deciphering this native control mechanism is underscored by recent work by Ju et al. and Weissensteiner et al., who successfully re-engineered *Cv*FAP to catalyze non-natural, asymmetric carbon–carbon bond-forming reactions.^13,47^ Crucially, unlocking these novel biosynthetic pathways required the disruption of the native solvent-mediated PCET network. A comprehensive understanding of how structural water networks dictate the fate of transient radical intermediates offers actionable design principles for expanding the synthetic repertoire of engineered photoenzymes.

## Supplementary Material









## Data Availability

The data underlying this study are openly available in Zenodo at 10.5281/zenodo.19484835

## Notes

Movie 1: Bicarbonate anion formation (AVI) (MP4)
Movie 2: Neutral R451^(0)^ formation via a direct proton transfer (AVI) (MP4)
Movie 3: Neutral R451^(0)^ formation via water-mediated proton transfer (AVI) (MP4)
Computational protocol; The photoinduced forward electron transfer; QM/AMOEBA MD on the diradical [FAD^•–^ R-COO^•^] scenario; MM MD on the [FAD^•–^ + R^•^ + CO_2_] scenario; MM MD on WT *Cv*FAP; Different scenarios for the R-H formation; Effect of the butterfly bending on the excitation energies of FAD; Additional figures and tables (PDF)
