# Supplementary material for: Molecular Mechanism of the Catalytic Radical Termination in Fatty Acid Photodecarboxylase
Source: J Am Chem Soc. 2026 Jul 8;148(28):30163–70. doi: 10.1021/jacs.6c07143 (PMC13397541; doi:10.1021/jacs.6c07143)
Supplement: Supplementary file 4 [file ja6c07143_si_004.pdf]

SUPPORTING INFORMATION OF

Molecular Mechanism of the Catalytic Radical Termination in Fatty Acid Photodecarboxylase

Giacomo Londi*[1] and Benedetta Mennucci*[1]

[1]Department of Chemistry and Industrial Chemistry, University of Pisa, 56124 Pisa, Italy

E-Mail: giacomo.londi@unipi.it, benedetta.mennucci@unipi.it

TABLE OF CONTENT

1) Computational protocol 2

2) The photoinduced forward electron transfer 7

3) QM/AMOEBA MD on the diradical [FAD•$^-$ R-COO•] scenario 8

4) MM MD on the [FAD•$^-$ + R• + CO_2] scenario 9

5) MM MD on WT *Cv*FAP 15

6) Different scenarios for the R-H formation 16

7) Effect of the butterfly bending on the excitation energies of FAD 20

8) Additional figures and tables 22

References 27

1) Computational protocol

The computational protocol presented in this work:

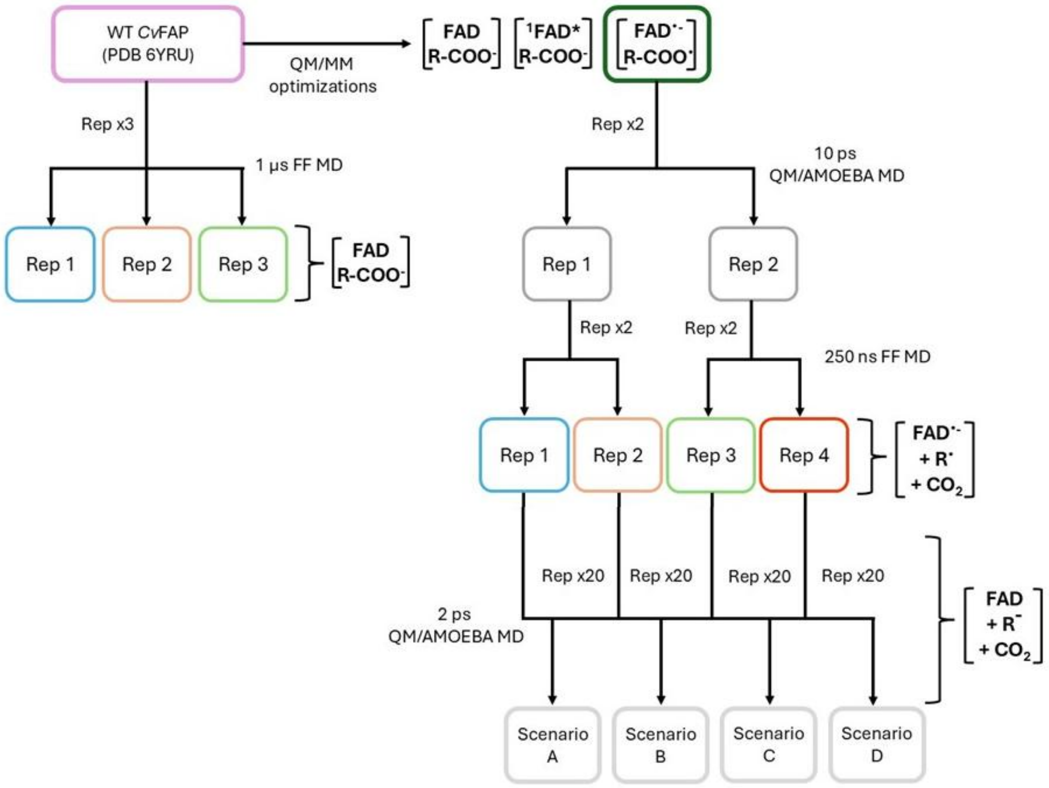

The crystallographic structure of Wild-Type (WT) *Cv*FAP (RSCB PDB entry: 6YRU)[1] was used as starting point for two series of simulations. We performed three 1-µs long MM MD replicas of the dark-adapted state (*i.e.*, before the photoexcitation) and we extracted the configurations used for the simulation of the absorption spectrum of FAD@*Cv*FAP. In the other series of calculations, we initially performed QM/MM optimizations of the three scenarios involved in the forward electron transfer process, namely [FAD R-COO$^-$], [^{1}FAD* R-COO$^-$], and [FAD$\bullet^-$ R-COO•]. The latter was used as starting point for two 10-ps long QM/MM MD replicas to observe the decarboxylation event. Then, we ran four 250-ns long MM MD simulations of the [FAD$\bullet^-$ + R• + CO_2] system, followed by 80 QM/MM MD simulations by implicitly assuming that FAD$\bullet^-$ has transferred back an electron to R•, to explore the pathways leading to the product R-H formation. The different scenarios were finally used to investigate the change in the absorption spectra of FAD.

MM MD simulations on *Cv*FAP

The crystallographic structure (RSCB PDB entry: 6YRU)[1] was used as starting point for classical force field-based MD simulations on *Cv*FAP. Protonation states were assigned with MCCE (Multi-Conformation Continuum Electrostatics)[2,3] software at pH 8.5, optimal for the photoenzyme's activity in solution, and histidine protonation states were further checked by visual inspection. The protein is electrically neutral, while the total charge of the system is -4, due to two deprotonated fatty acids (*e.g.*, stearic acids, C18, as found in the crystal structure) and the $[FAD]^{2-}$ cofactor. Potassium counterions were added to neutralize the system. Missing hydrogen atoms were introduced with the *leap* tool available in Amber22.[4] The oxidized flavin core and the fatty acid (FA) were optimized at the MP2 level with the cc-pVDZ basis set, in conjunction with a polarizable continuum model (PCM)[5] in water. Their RESP charges were obtained at the MP2/*aug*-cc-pVDZ level of theory in PCM and by using the *antechamber* tool available in Amber22.[4]

All the residues were described with the ff15ipq implicitly polarized force field,[6] combined with the SPC/E model for waters,[7] while the FA with the general Amber force field.[8] The parameters for the FAD species were obtained by taking the literature flavin mononucleotide (FMN) parameters,[9] and completing them with the ones of the adenosine diphosphate (ADP).[10] The *Cv*FAP system was solvated within a truncated octahedron box with a cutoff of 18 Å from the protein edges. All the water molecules and the protein backbone were subjected to molecular mechanics (MM) energy minimizations, with the first 2500 steps of steepest descent and the rest of conjugate gradient until convergence. The movement of the protein backbone was initially restrained by applying a 10 kcal mol^{-1} Å^{-2} harmonic potential. Then, the system was heated up to 298 K in the NVT ensemble for 2 ns, followed by a total of 3 ns-long equilibration steps in the NPT ensemble, where the restraint on the protein backbone was gradually released. Finally, in the NPT production run three independent replicas were propagated without any restraint for 1 μs, saving coordinates every 50 ps, and discarding the first 200 ns from the structural and electronic properties analysis.

QM/AMOEBA MD simulations

In all QM/MM MD simulations we used the polarizable force field AMOEBA,[11,12] combined with the DFT level of theory. Two sets of trajectories were generated: one to observe the decarboxylation and the other to produce the different scenarios yielding the product R-H. In all cases, the size of the system was reduced by keeping a shell of water molecules around 12 Å from the protein edges, where the outer 8 Å sphere was kept frozen during the simulations. After minimization, the QM/AMOEBA MD trajectories were propagated in the NVT ensemble using the Bussi thermostat at room temperature (300 K),[13] with a time constant of 0.1 ps, and an integration step of 0.5 fs.

To run all QM/AMOEBA MD simulations, we used the machinery developed in our group by interfacing Tinker,[14] working as MD engine and computing the bonded and van der Waals terms of the energy and forces, with a locally modified version of the Gaussian suite of programs,[15]

which computes the QM/AMOEBA energy, forces, and electrostatic and polarization contributions. We also took advantage of the quasi time-reversible scheme based on Grassmann extrapolation of density matrices to reduce the number of self-consistent field iterations, while achieving energy-conserving simulations.[16]

MM MD on the [FAD•⁻ + R• + CO_2] scenario

As a starting point for these MD simulations, we used a frame extracted from the two QM/AMOEBA MD trajectories after the decarboxylation took place. To define the MM parameters of the anionic semiquinone flavin core, the alkyl radical, and CO_2 species we performed geometry optimization at the MP2 level with the cc-pVDZ basis set, in conjunction with a polarizable continuum model (PCM)[5] in water. The RESP charges of each species were obtained at the MP2/*aug*-cc-pVDZ level of theory in PCM and by using the *antechamber* tool available in Amber22.[4]

All the residues were described with the ff15ipq implicitly polarized force field,[6] combined with the SPC/E model for waters,[7] while the alkyl radical and CO_2 with the general Amber force field.[8] The systems were solvated within a truncated octahedron box with a cutoff of 18 Å from the protein edges. All the water molecules and the protein backbone were subjected to molecular mechanics (MM) energy minimizations. The systems were then heated to 298 K for 2 ns in the NVT ensemble applying a constraint of 10 kcal mol^{-1} $Å^{-2}$ on the whole enzyme. Then, we performed 3 ns of NPT equilibration steps, gradually removing the 10 kcal mol^{-1} $Å^{-2}$ harmonic restraint imposed on the protein backbone. Finally, four independent replicas were produced for 250 ns in the NPT ensemble, saving coordinates every 50 ps. All MD simulations were performed with Amber22, applying the particle mesh Ewald (PME) truncation method (with a short-range cut-off of 10 Å), an integration step of 2 fs, the SHAKE algorithm, a Langevin thermostat with a friction coefficient of 1 ps^{-1}, and the Monte Carlo barostat for NPT simulations.

Electronic excitation calculations

Frames extracted from either fully MM or QM/AMOEBA MD trajectories were additionally subjected to DFT optimization within the electrostatic embedding QM/MM scheme,[17] as implemented in the Gaussian16 suite of programs.[18] In such calculations, only the flavin's isoalloxazine ring was optimized at the ωB97X-D/6-31+G(d) level of theory to suppress fluctuations in the internal coordinates due to thermal motions. On top of the optimized structures, single-point energy calculations were performed by using the TD-DFT/AMOEBA polarizable embedding scheme.[19] In the choice of the DFT functional we opted for the optimally tuned (OT) ωB97X-D, where the range-separation parameter ω was non-empirically "gap-tuned" for the flavin core,[20,21] and set to $\omega = 0.160$ $Bohr^{-1}$, in conjunction with the 6-31+G(d,p) basis set. Additionally, TD-DFT/AMOEBA calculations were run by using two different exchange-correlation functionals: CAM-B3LYP and B3LYP.

- FAD@*Cv*FAP: we used 120 frames, extracted from the three MM MD replicas of the dark-adapted state. For TD-DFT/AMOEBA calculations, the QM region included the flavin core, the deprotonated FA, the sidechain of R451, C432, and Y466;
- Scenario A: we used 130 frames, extracted from the 12 biased QM/AMEOBA MD trajectories of scenario A (prolonged to 10 ps, extracting frames every 1 ps) and from the only unbiased QM/AMOEBA MD trajectory of scenario B (prolonged to 10 ps, extracting frames every 1 ps), which gave bicarbonate formation. For TD-DFT/AMOEBA calculations, the QM region included the flavin core, the product R-H, the bicarbonate anion HCO_3^-, the sidechain of R451, C432, and Y466;
- Scenario B: we used 90 frames, extracted from the 9 unbiased QM/AMOEBA MD simulation of scenario B (prolonged to 10 ps, extracting frames every 1 ps). For TD-DFT/AMOEBA calculations, the QM region included the flavin core, the product R-H, CO_2, the sidechain of R451$^{(0)}$, C432, and Y466.

Absorption spectra

To simulate the absorption spectra corresponding to the lowest-energy band in the flavin we adopted a strategy which combines the inhomogeneous broadening resulting from a set of different configurations extracted from an MD trajectory and the vibronic features through the spectral density formalism. Spectral densities were calculated from the normal mode analysis, which assumes that the potential energy surface of the ground and the excited state is described by the same harmonic potential, but with a shifted equilibrium (displaced harmonic oscillator formalism,). Within this framework, the spectral density can be computed as:

$$J(\omega) = \pi \sum_k \omega_k \lambda_k \delta(\omega - \omega_k)$$

where ω_k and λ_k are, respectively, the frequency and the reorganization energy along the k-th normal mode. The reorganization energy along each normal mode is determined in terms of the excited-state gradient at the Franck-Condon point (vertical gradient). Here, we used the crystallographic structure of WT *Cv*FAP and, upon optimization of the ground state flavin core within the electrostatic embedding QM/MM scheme, we computed the flavin normal modes at the DFT OT ωB97X-D/6-31+G(d) level of theory and the first excited-state vertical gradients along theses normal modes at the TD-DFT OT ωB97X-D/6-31+G(d) level of theory. The computed spectral density is assumed to be identical for FAD @*Cv*FAP and all the different scenarios. The final spectra are computed by summing the contribution of N configurations of the system with excitation energies ω_{01}(j) and transition dipoles μ_{01}(j):

$$A(\omega) = \omega \frac{1}{N} \sum_{j=1}^{N} |\mu_{01}(j)|^2 S(\omega - \omega_{01}(j))$$

where S is the homogeneous lineshape calculated at the selected temperature from the spectral density J(ω) within the second-order cumulant expansion in the displaced harmonic oscillator formalism (see details in Ref. [22]).

Electronic couplings

Electronic couplings were computed within the frontier orbital approximation,[23,24] at the QM/AMOEBA. The chosen QM level of theory was ωB97X-D/6-31G(d). The same approach was used for the photoinduced forward ET between the FA's carboxylate group and the excited FAD and for the back ET between R• and $FAD•^-$. In the latter case, we used 250 frames extracted at each 1 ns of the four replicas of the [$FAD•^-$ + R• + CO_2] system.

2) The photoinduced forward electron transfer

In our previous work[25] we used a different R451 rotamer with respect to the one found in the crystallographic structure. Here, we repeated all the calculations with the crystallographic R451 rotamer (see Figure 1a-b in the main text) applying the same computational protocol. We found that: i) at the optimized [^{1}FAD* R-COO$^-$] geometry, the energy of the locally excited (LE) state is 3.02 eV (*vs* 2.99 in the previous study); ii) at its minimum geometry, the charge transfer (CT) state [FAD•$^-$ R-COO•] is 2.84 eV (*vs* 2.70 eV in the previous study) thus giving LE-CT energy difference $\Delta E = -0.18\ eV$. We also recalculated the reorganization energy $\lambda = 1.06\ eV$ and the electronic coupling V_{ET} between the substrate and the excited flavin at the optimized [^{1}FAD* R-COO$^-$] geometry. The V_{ET} is 40 meV, which is almost twice larger as the V_{ET} computed in the previous study (*vs* 23 meV). By using the same analysis used in the previous work, we estimated the coupling to be used in the semiclassical Marcus theory at the crossing between the LE-CT potential energy surface (see Figure S1), obtaining a V_{ET} of ~32 meV. By inserting the new parameters into the Marcus equation we obtained a rate of $\kappa_{fET} = 1.5x10^{10}\ s^{-1}$, yielding a timescale of ~70 ps.

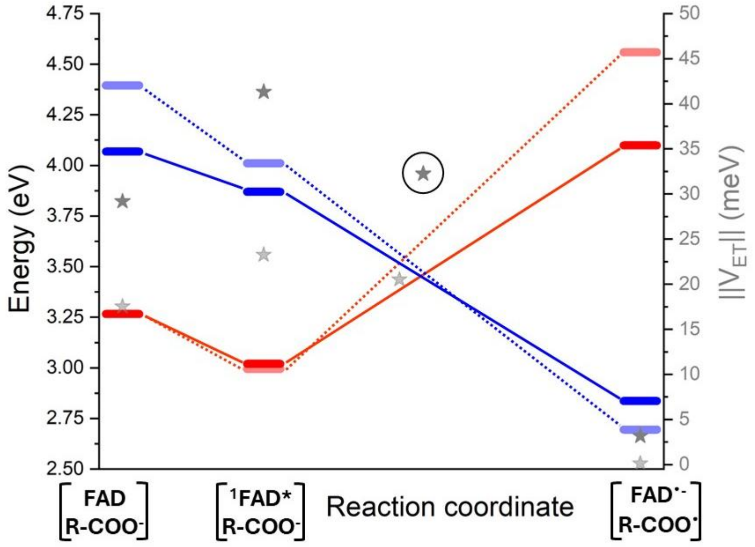

Figure S1: Locally-excited (LE, in red) and charge-transfer (CT, in blue) energies at the geometries optimized for [FAD R-COO$^-$], [^{1}FAD* R-COO$^-$], and [FAD•$^-$ R-COO•]. The absolute value (in meV) of the electronic couplings V_{ET} (gray stars) are also reported in the right y-axis. Shaded bars and stars refer to results obtained in Ref. 23 for a different R451 rotamer, while solid bars and stars refer to this current work. In particular, the circled gray star indicates our estimation of V_{ET} at the supposedly LE-CT crossing.

3) QM/AMOEBA MD on the diradical [FAD•⁻ R-COO•] scenario

We also performed two independent QM/AMOEBA MD replicas starting from the optimized diradical [FAD•⁻ + R-COO•] system, as shown in Figure 1b (in Figure S2 we show two representative frames). Here, we chose as QM region only the radical R-COO•, that was treated at the unrestricted DFT ωB97X-D/6-31G(d) level of theory, while the environment by the polarizable AMOEBA force field, having parametrized charges and quadrupoles for the anionic semiquinone FAD•⁻ species. In both replicas the decarboxylation took place after 2 ps, thus suggesting an even lower energy barrier of ~2 kcal mol^{-1}, than previously estimated.[25]

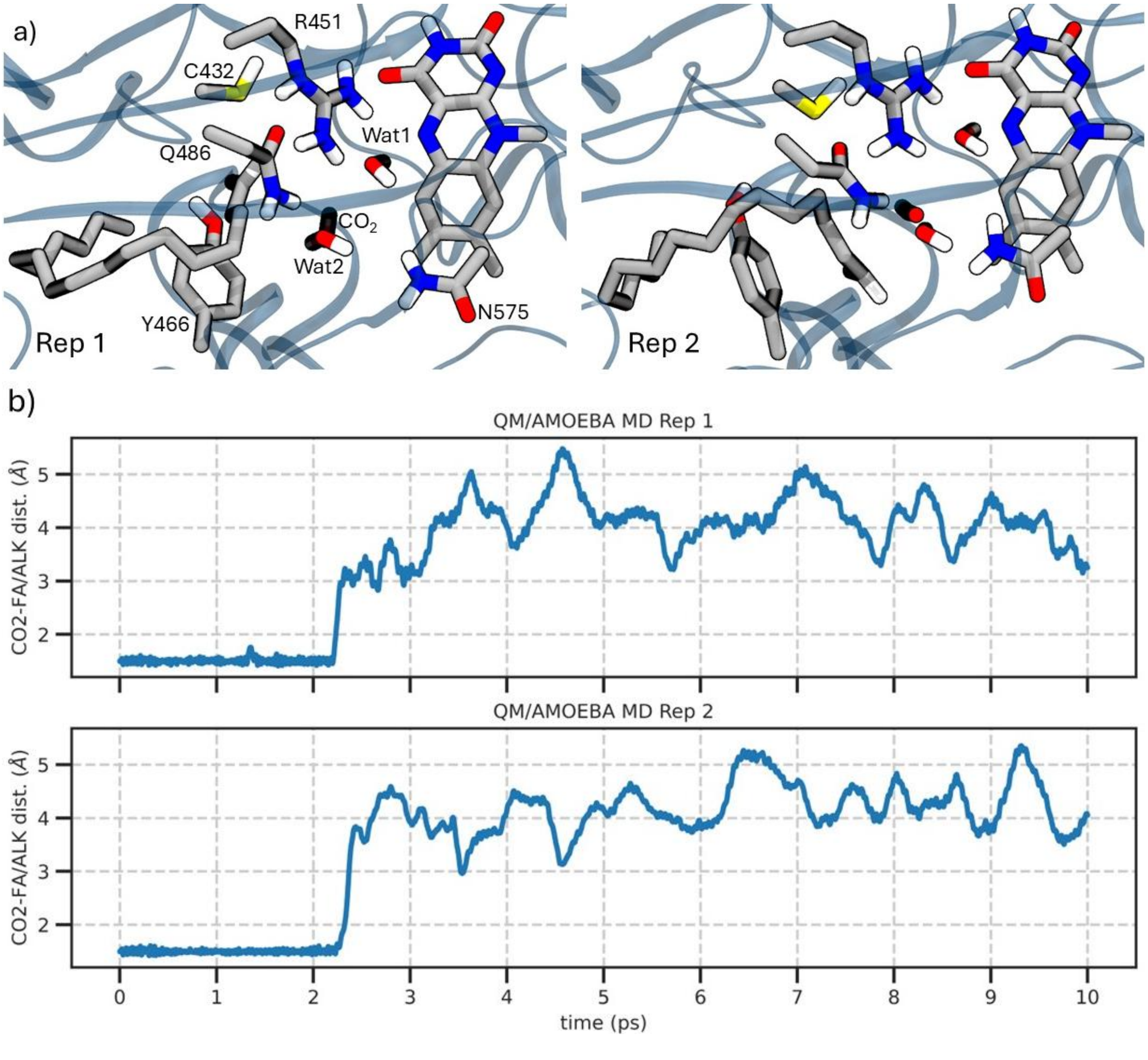

Figure S2: Structure of the active site in frames extracted from the two QM/AMOEBA MD trajectories, highlighting CO_2 release from the fatty acid (FA, panel a); the length of the C-C bond involved in the cleavage as a function of the QM/AMOEBA MD simulation time (panel b).

4) MM MD on the [FAD•$^{-}$ + R• + CO_2] system

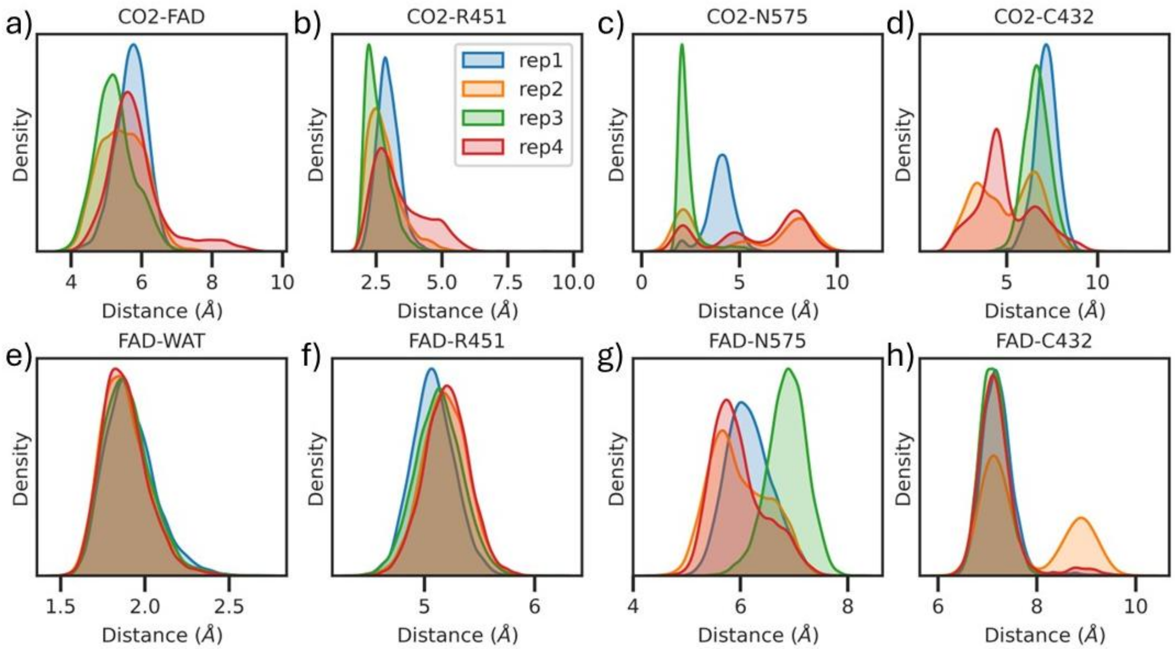

Figure S3: Distributions of minimum distances between CO_2 and the N5 atom of FAD•$^{-}$ (panel a, see also Figure S8) or the amino acid sidechains heteroatoms' hydrogens (panel b-d); between the N5 atom of FAD•$^{-}$ and: the water hydrogens (panel e); the sidechain nitrogen atoms in R451 (panel f); the sidechain nitrogen atom in N575 (panel g); the sidechain sulfur atom in C432 (panel h).

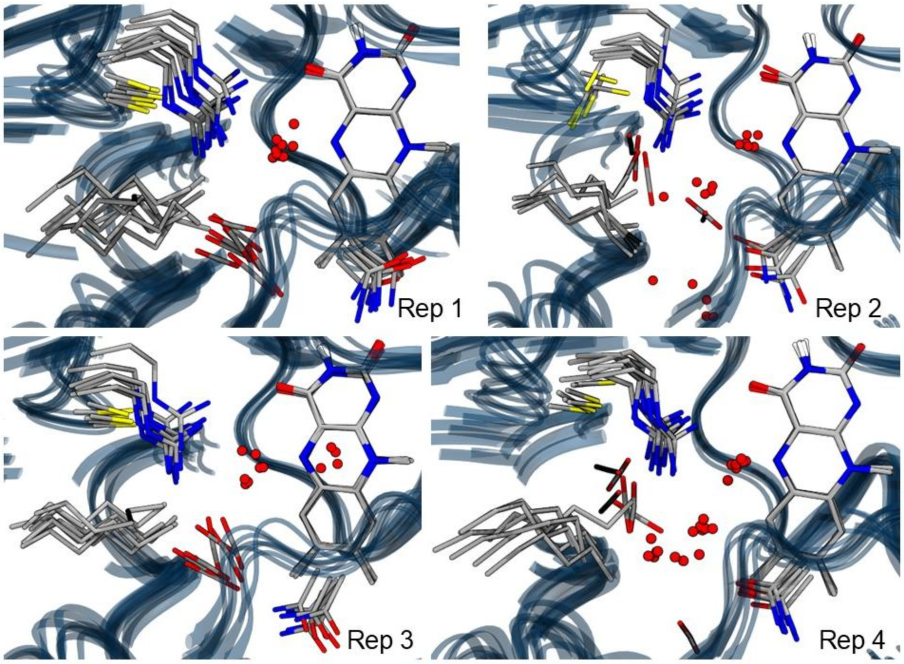

Figure S4: Superimposition of 40 initial structures of the QM/AMOEBA MD trajectories obtained by the four MM MD replicas of the [FAD•$^-$ + R• + CO_2] system. The structures are aligned on the flavin isoalloxazine ring. Water molecules either within 3 Å from the N5 atom of FAD or within 5 are Å from the alkyl radical R• are represented by red dots, while the sidechains of R451, C432, and N575 are shown as sticks, as well as only the first six carbon atoms of the alkyl radical. Hydrogens are removed for clarity.

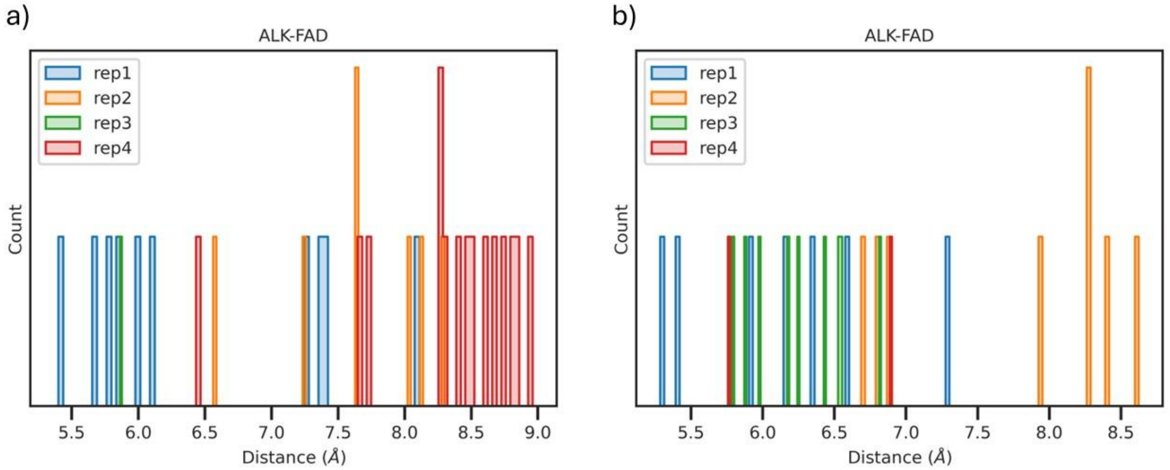

Figure S5: Histograms of minimum distances between the alkyl sp^2 carbon-radical centered R'-H_2C• and the N5 atom of FAD for the initial frames of the QM/AMOEBA trajectories that produced scenario B, either involving as proton donor a water molecule (panel a: 10 rep1, 7 rep2, 1 rep3, 15 rep4) or the protonated R451 (panel b: 7 rep1, 8 rep2, 10 rep3, 2 rep4).

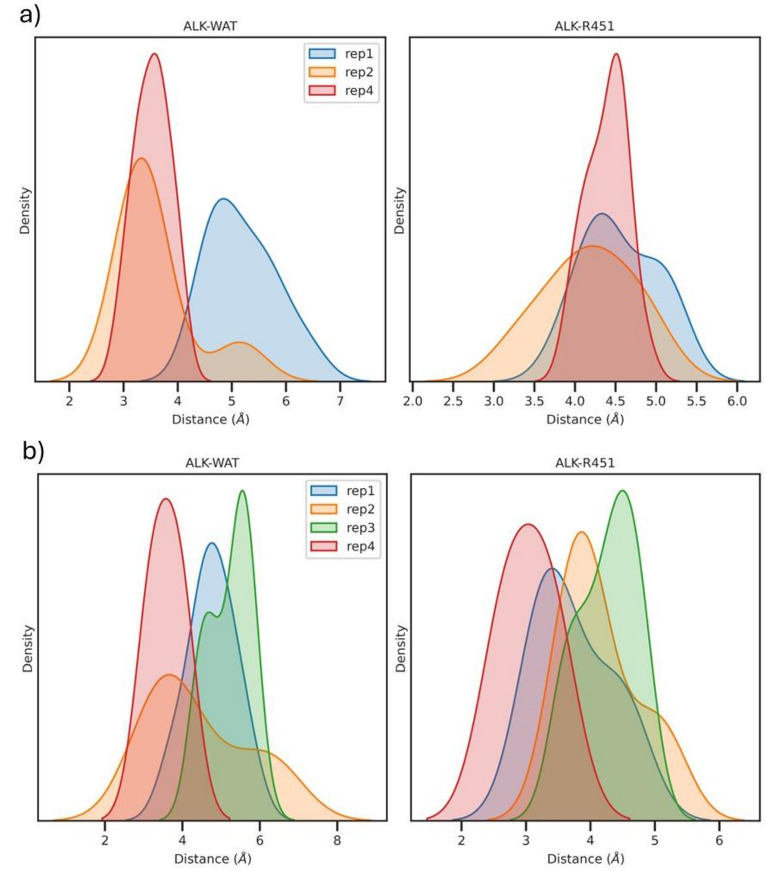

Figure S6: Distributions of minimum distances between the alkyl sp^2 carbon-radical centered R'-H_2C• and the water hydrogens or the guanidinium group's hydrogens for the initial frames of the QM/AMOEBA trajectories that produced scenario B, either involving as proton donor a water molecule (panel a: 10 rep1, 7 rep2, 15 rep4) or the protonated R451 (panel b: 7 rep1, 8 rep2, 10 rep3, 2 rep4).

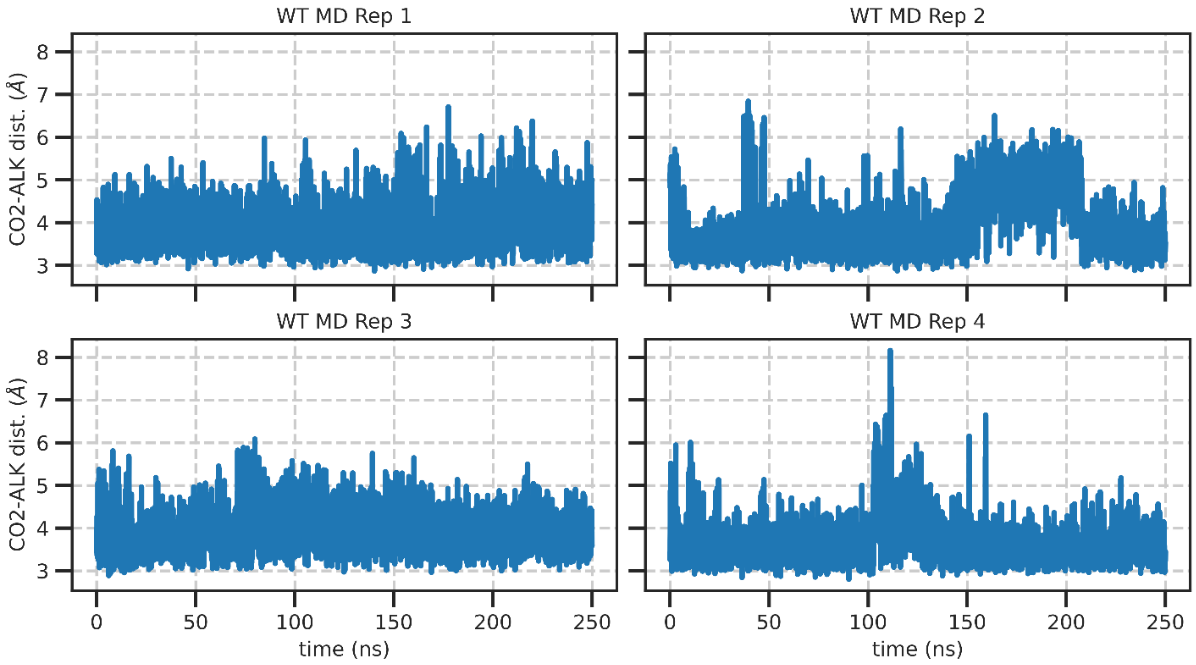

Figure S7: The distance between CO_2 and the alkyl sp^2 carbon-radical centered R'-H_2C• as a function of the simulation time for the four replicas of the [FAD•$^-$ + R• + CO_2] system.

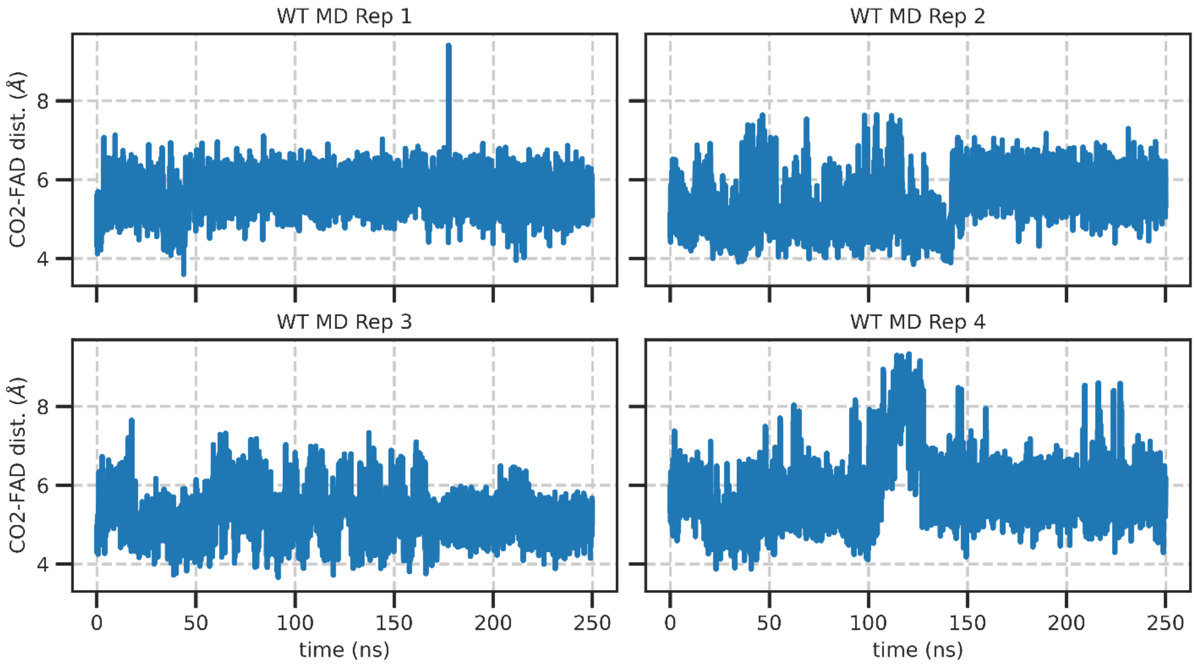

Figure S8: The distance between CO_2 and the N5 atom of FAD as a function of the simulation time for the four replicas of the [FAD•$^-$ + R• + CO_2] system.

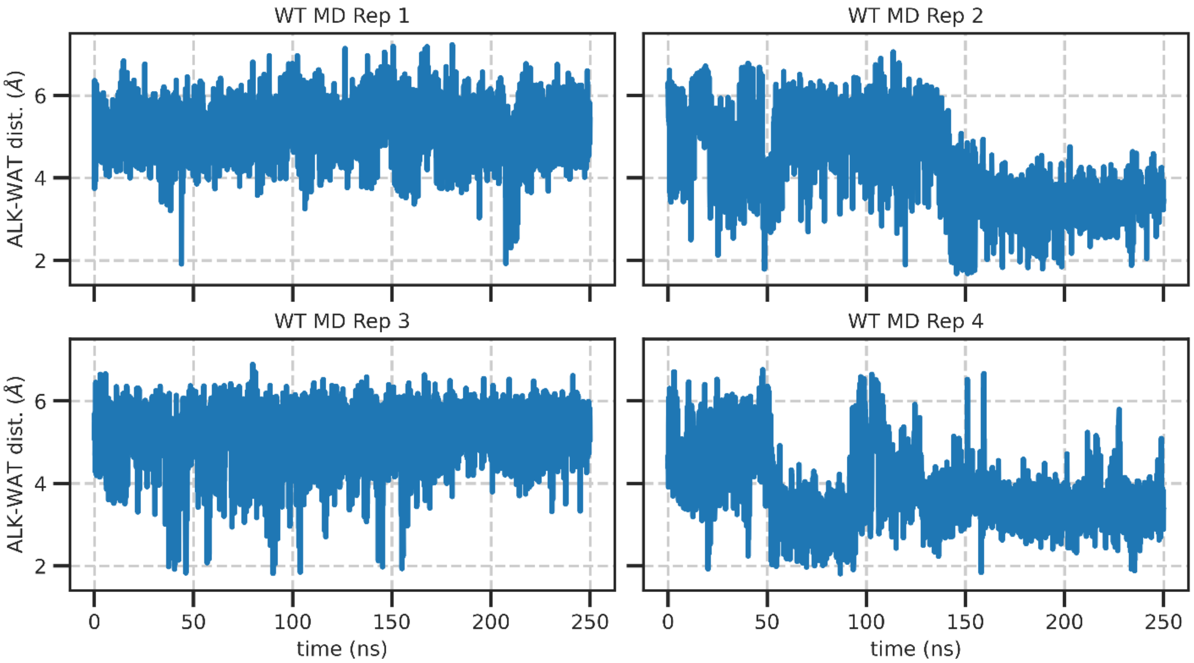

Figure S9: The distance between the alkyl sp^2 carbon-radical centered R'-H_2C• and the water hydrogen as a function of the simulation time for the four replicas of the [FAD•$^-$ + R• + CO_2] system.

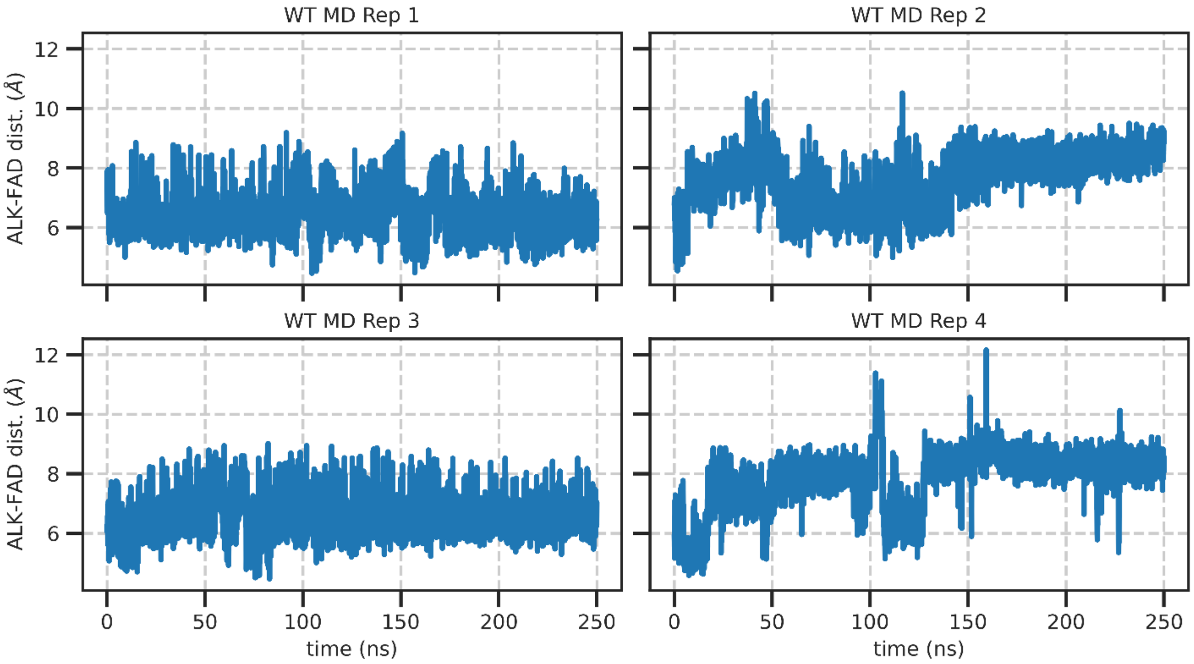

Figure S10: The distance between the alkyl sp^2 carbon-radical centered R'-H_2C• and the N5 atom of FAD as a function of the simulation time for the four replicas of the [FAD•$^-$ + R• + CO_2] system.

To support the hypothesis for which the bET from FAD•⁻ to R• is the driving force in the PT mechanism, we calculated the electronic couplings between the two radical species for 250 frames extracted (every 1 ns) from the four replicas of the [FAD•⁻ + R• + CO_2] system. The resulting distributions reflect the distances distribution shown in Figure 2c. In replica 1 and 3, where R• and FAD•⁻ are closer, an average value of ~30 meV was found for the coupling, which reduces to ~20 meV in replica 2 and 4, where the two radical species are farther apart.

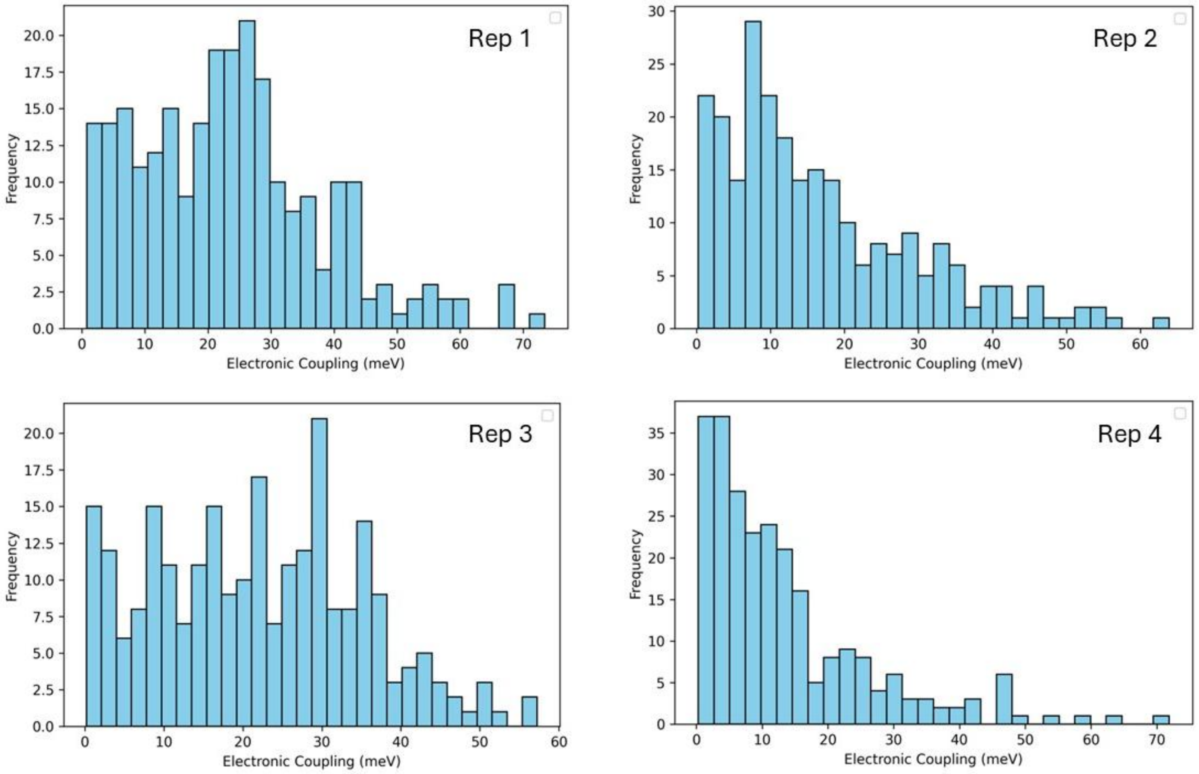

Figure S11: Distributions of the absolute value of the electronic coupling (in meV) between the alkyl radical R• and FAD•⁻ for the four replicas of the [FAD•⁻ + R• + CO_2] system.

5) MM MD on the WT *Cv*FAP system

The three replicas show similar distributions in the fatty acid (FA)-FAD distances with a maximum peak at ~3.5 Å, suggesting a close interaction between the two molecules, and a long tail extending >5 Å, indicating that there are few cases when the FA moves away from the flavin core. As observed also in the crystal structure, during the MD simulation the FA forms a stable H-bond network with R451 (80% of the time), N575 (86% of the time), and water molecules around. The N5 atom of FAD interacts through a H-bond mostly with A171 (28%) and rarely with a water molecule, consistent with previous observations.[26]

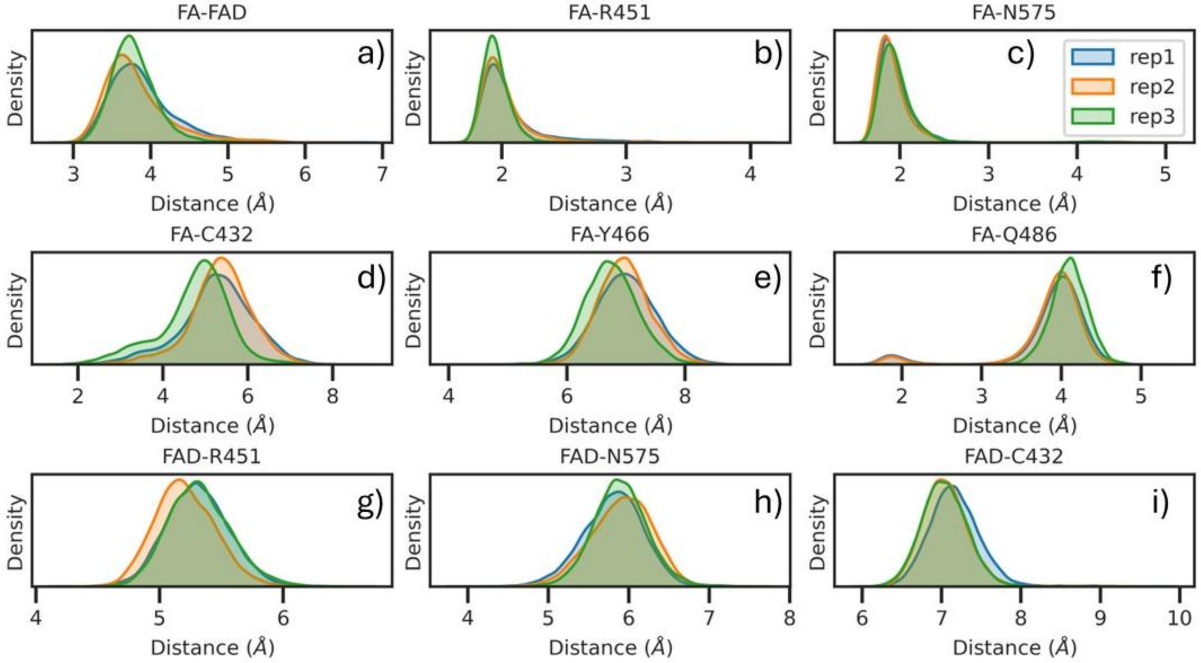

Figure S12: Distributions of the minimum distances between the fatty acid's (FA) carboxylate group and: the N5 atom of FAD (panel a); the sidechain nitrogen atoms in R451 (panel b); the sidechain nitrogen atom in N575 (panel c); the sidechain sulfur atom in C432 (panel d); the sidechain oxygen atom in Y466 (panel e); the sidechain nitrogen atom in Q486 (panel f). Distributions of the minimum distances between the N5 atom of FAD and: the sidechain nitrogen atoms in R451 (panel g); the sidechain nitrogen atom in N575 (panel h); the sidechain sulfur atom in C432 (panel i).

6) Different scenarios for the product R-H formation

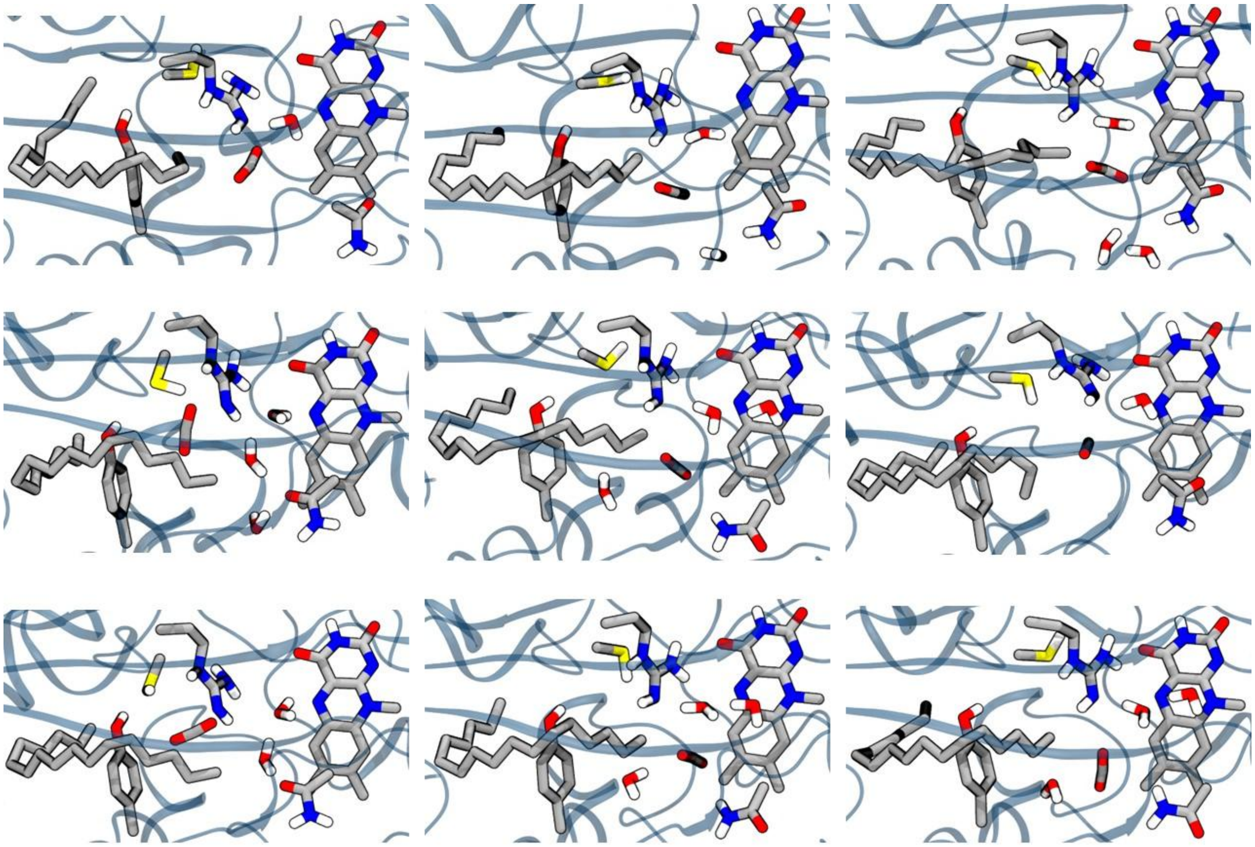

Figure S13: Examples of structures yielding scenario B: R451 directly donating a proton to R^-.

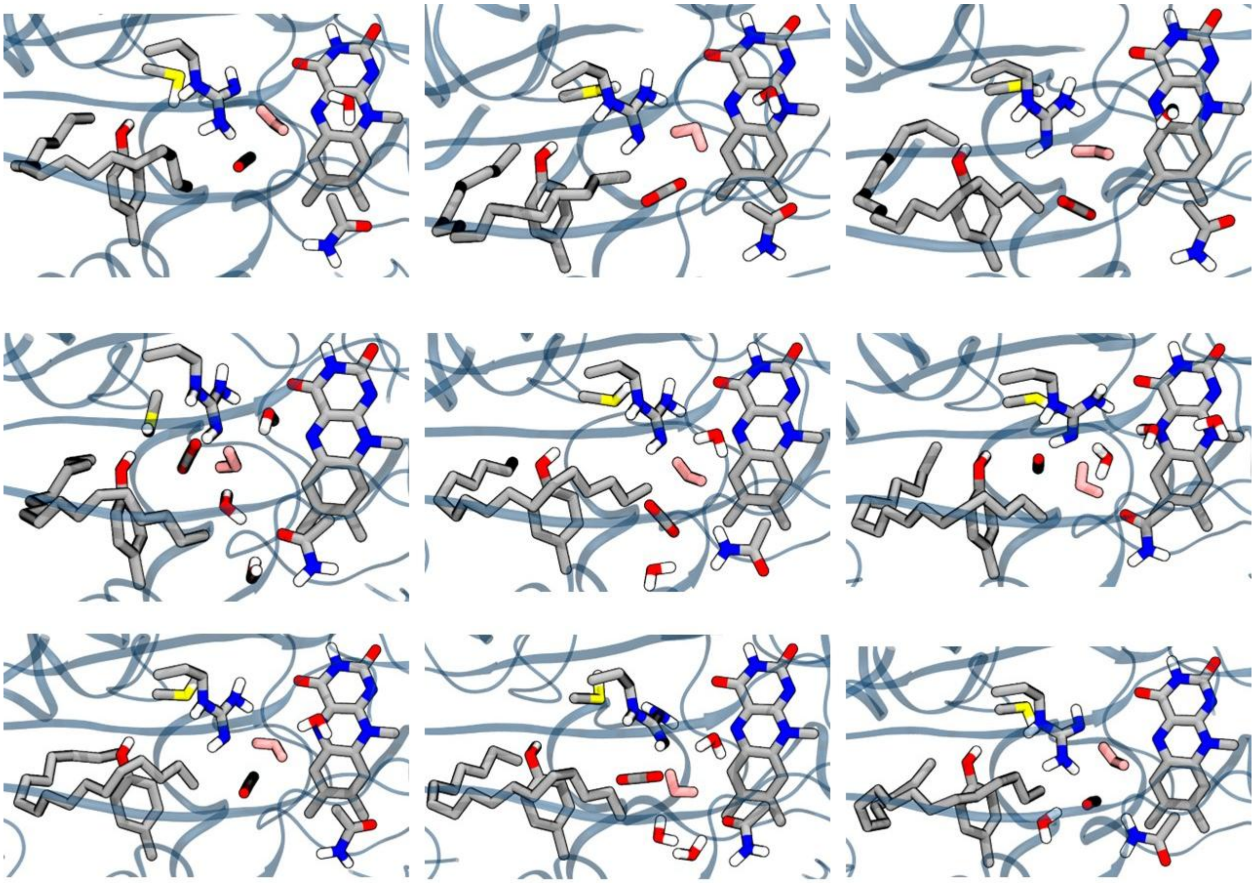

Figure S14: Examples of structures yielding scenario B: the water molecule (in pink) involved in the PT to R^- and from R451.

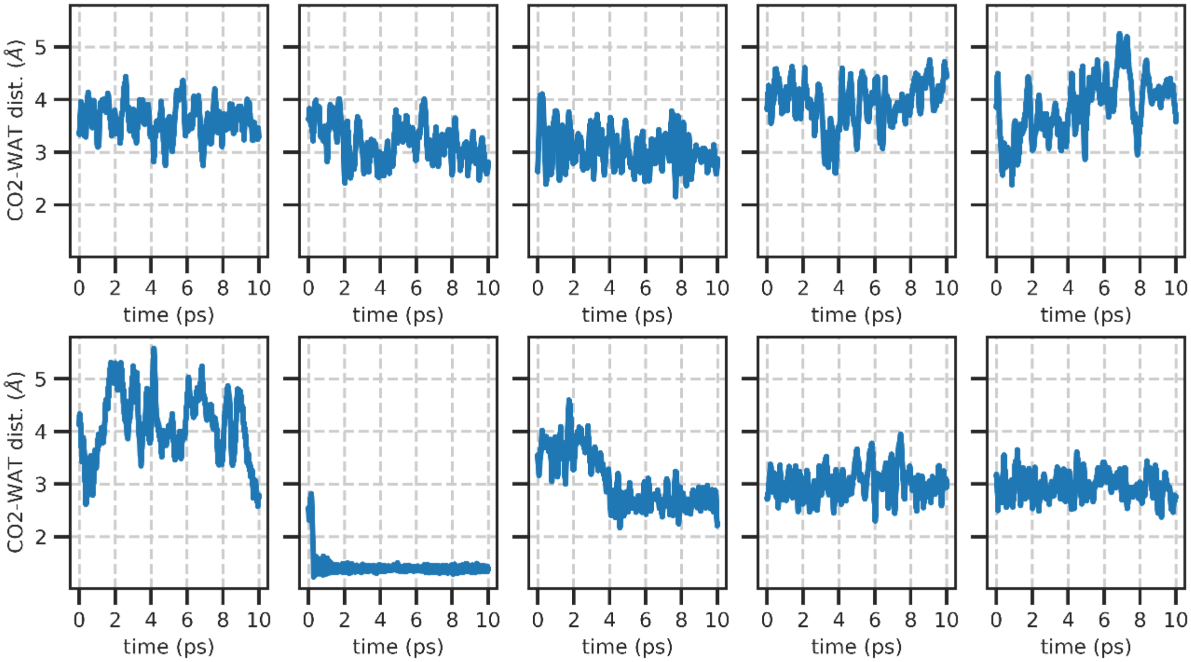

Figure S15: The minimum distance between CO_2 and water oxygen as a function of the simulation time for the extended 10 QM/AMOEBA MD trajectories of scenario B. Here, only one trajectory evolved towards the formation of HCO_3^-.

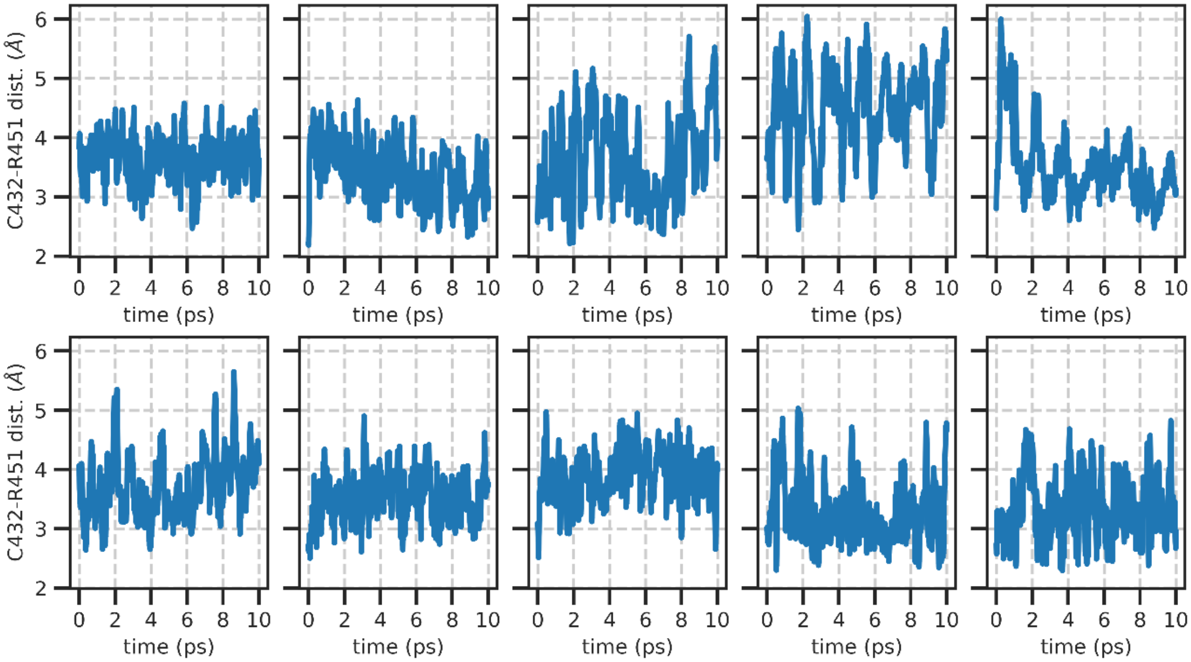

Figure S16: The minimum distance between the hydrogen of the C432 thiol group and the nitrogen atoms of the R451 guanidinium group as a function of the simulation time for the extended 10 QM/AMOEBA MD trajectories of scenario B.

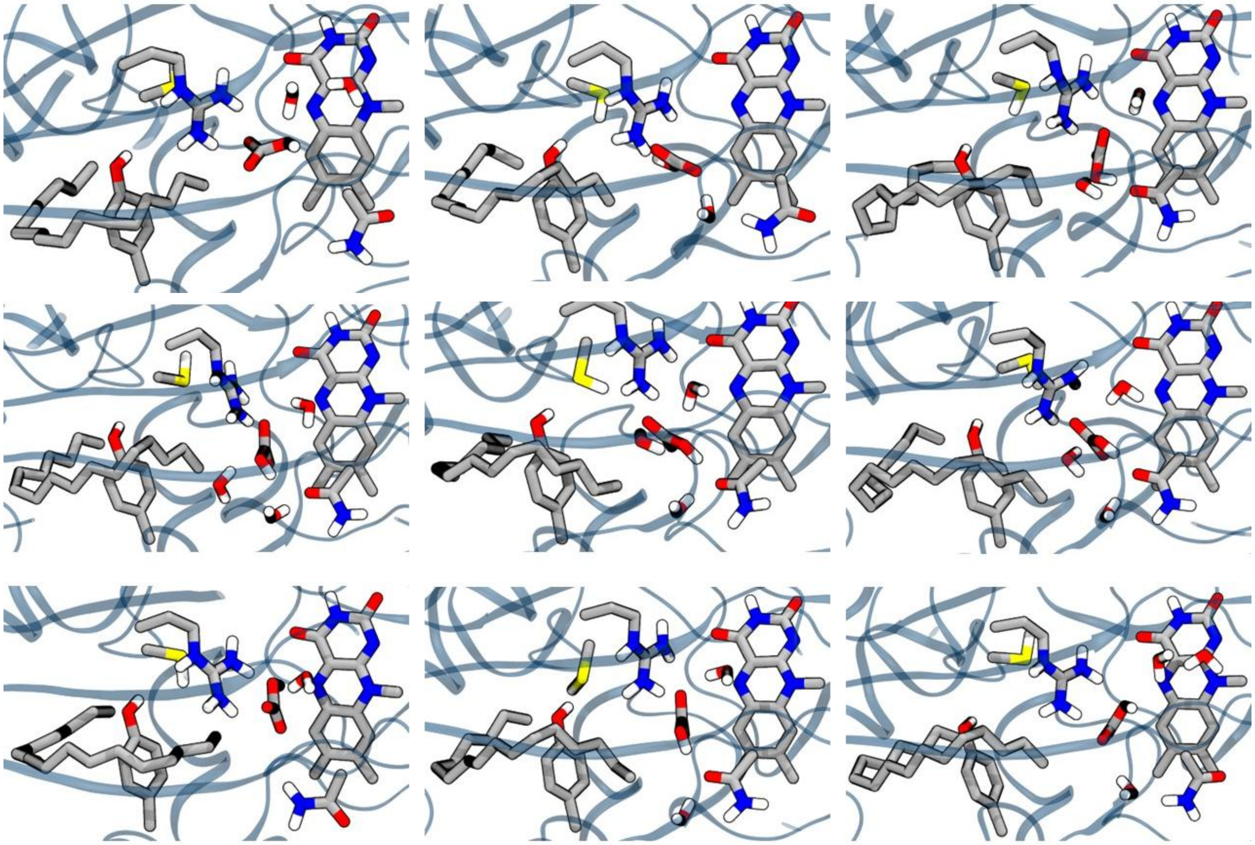

Figure S17: Examples of structures yielding scenario A: HCO_3^- formation via a water molecule donating a proton to R^-.

7) Effect of butterfly bending on the excitation energies of FAD

In *Cv*FAP the ground-state FAD absorption spectrum is redshifted by ~20 nm with respect to what observed in most flavoproteins, showing fully planar oxidized flavin core conformations.[27–30] This has implications in increasing the light-harvesting ability of *Cv*FAP and in the fET from the substrate, made more efficient by the flavin bending, which in turn lowers the CT state energy. Our TD-DFT/AMOEBA calculations on the FAD @*Cv*FAP show that such redshift is only partially induced by the conformation of the flavin isoalloxazine ring, with its butterfly angle kept bent at ~15-20° by the surrounding amino acids in *Cv*FAP. In fact, if we compare the excitation energies of FAD @*Cv*FAP with those obtained by keeping the same geometry for FAD but removing the substrate, the protein, and the solvent (*i.e.*, isolated), we see that the electrostatic effect of the whole surrounding environment contributes the most, red-shifting by ~18 nm the isolated FAD excitation energy (382 *vs* 400 nm for FAD @*Cv*FAP, see Figure S18). Moreover, while for the isolated FAD there exists a correlation between the bending of its butterfly angle and its excitation energy (*i.e.*, the more planar, the more blue-shifted), when FAD is embedded in the active site such correlation is lost, buried by the electrostatic disorder of the environment. Such trend is conserved even when we analyzed the structures coming from scenario A and B, namely calculating the FAD excitation energy in gas-phase and when embedded in the different protein environment (see Figure S19 and S20, respectively). In all the three scenarios, the butterfly bending angle of the flavin isoalloxazine ring keeps its conformation at ~20°.

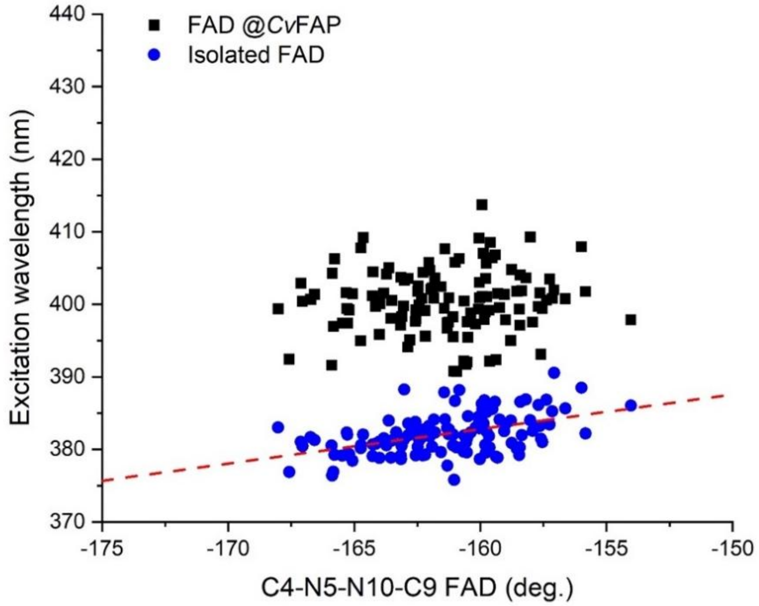

Figure S18: FAD excitation wavelength as a function of the butterfly bending angle (-161° ± 3°) in gas-phase (isolated FAD, in blue, 382 ± 3 nm) and @*Cv*FAP (in black, 400 ± 4 nm). The linear fit is highlighted with the dashed red line, with this set of parameters: R^2 = 0.22 and Pearson's correlation coefficient = 0.48. Number of data points: 120.

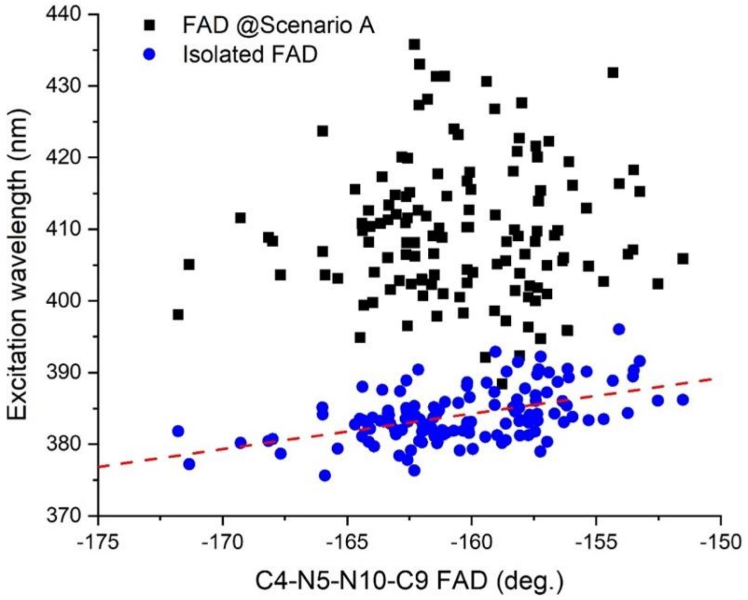

Figure S19: FAD excitation wavelength as a function of the butterfly bending angle (-160° ± 4°) in gas-phase (isolated FAD, in blue, 384 ± 4 nm) and FAD @ scenario A (in black, 410 ± 10 nm). The linear fit is highlighted with the dashed red line, with this set of parameters: R^2 = 0.25 and Pearson's correlation coefficient = 0.50. Number of data points: 130.

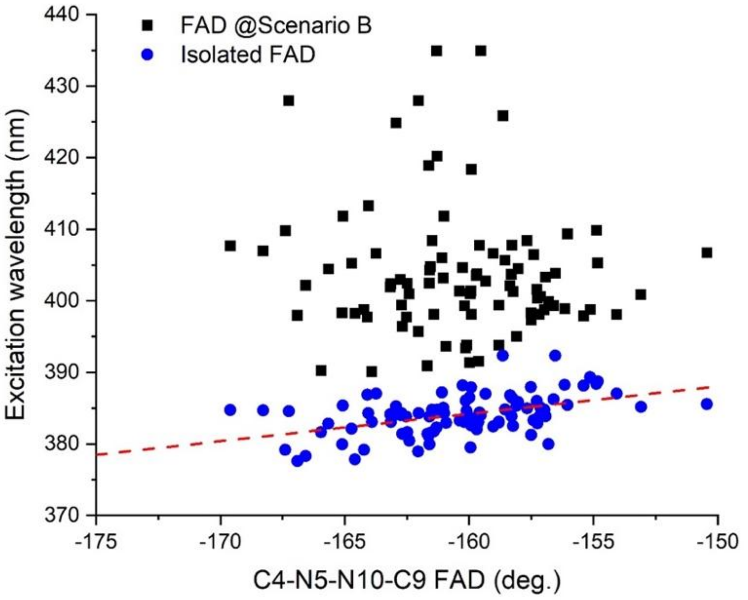

Figure S20: The FAD excitation wavelength as a function of the butterfly bending angle (-160° ± 4°) either in gas-phase (isolated FAD, in blue, 384 ± 3 nm) and FAD @ scenario B (in black, 404 ± 9 nm). The linear fit is highlighted with the dashed red line, with this set of parameters: R^2 = 0.21 and Pearson's correlation coefficient = 0.47. Number of data points: 90.

8) Additional figures and Tables

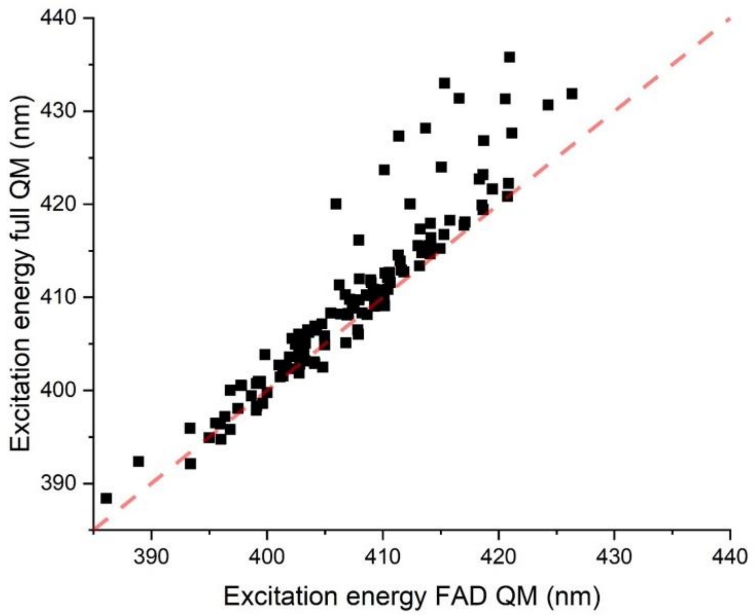

Figure S21: The correlation plot between the FAD excitation energy when the QM region comprised the flavin core, the bicarbonate anion HCO_3^-, the product R-H, the sidechain of R451, C432, and Y466 (*y* axis) and when only the flavin core was treated at the QM level (*x* axis). Level of theory: OT ωB97X-D/6-31+G(d) TD-DFT/AMOEBA.

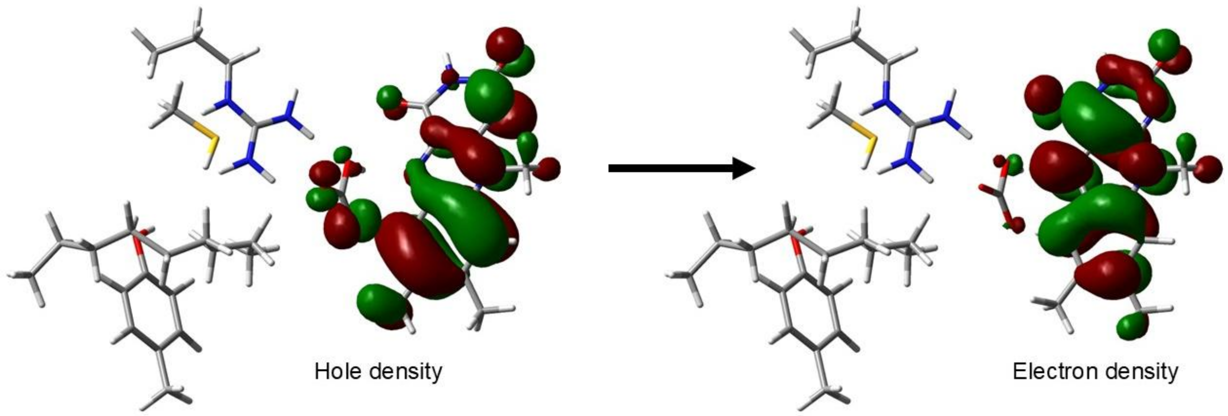

Figure S22: Natural transition orbitals (NTOs) for a structure in scenario A, where HCO_3^- forms an H-bond with the N5 atom of FAD and the hole-electron densities show an explicit contribution of the bicarbonate anion.

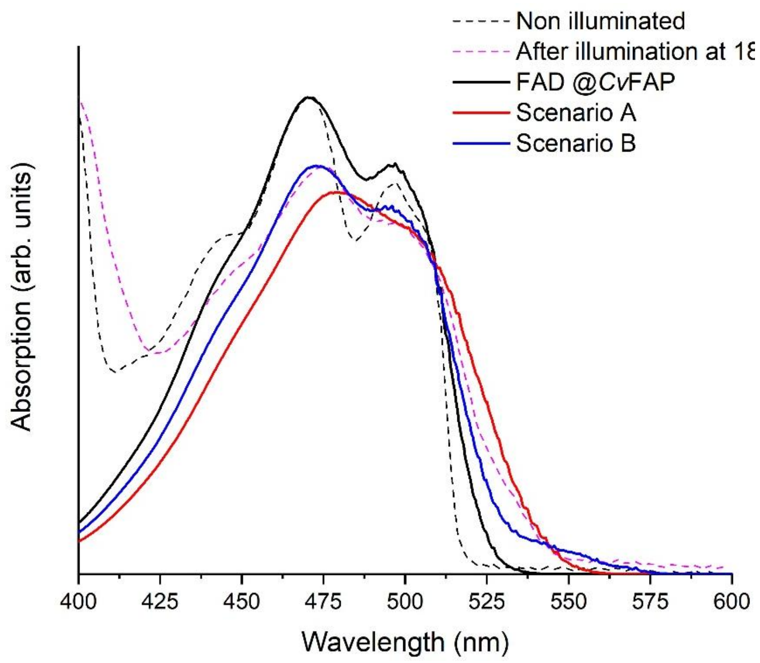

Figure S23: Experimental absorption spectra (black and magenta dotted lines, digitalized from Figure 2A in Ref.[31]) in comparison with the calculated lowest-energy band of the FAD @*Cv*FAP (black solid line) and as obtained by scenario A (red) and B (blue). Our simulated spectra were computed at cryogenic temperatures (both at 77 and 150 K), and since they are almost identical, only those obtained at 150 K are shown. The calculated FAD @*Cv*FAP spectrum was shifted by 0.41 eV (62 nm) in order to match the experimental one, and the calculated spectra of scenario A and B were consistently shifted.

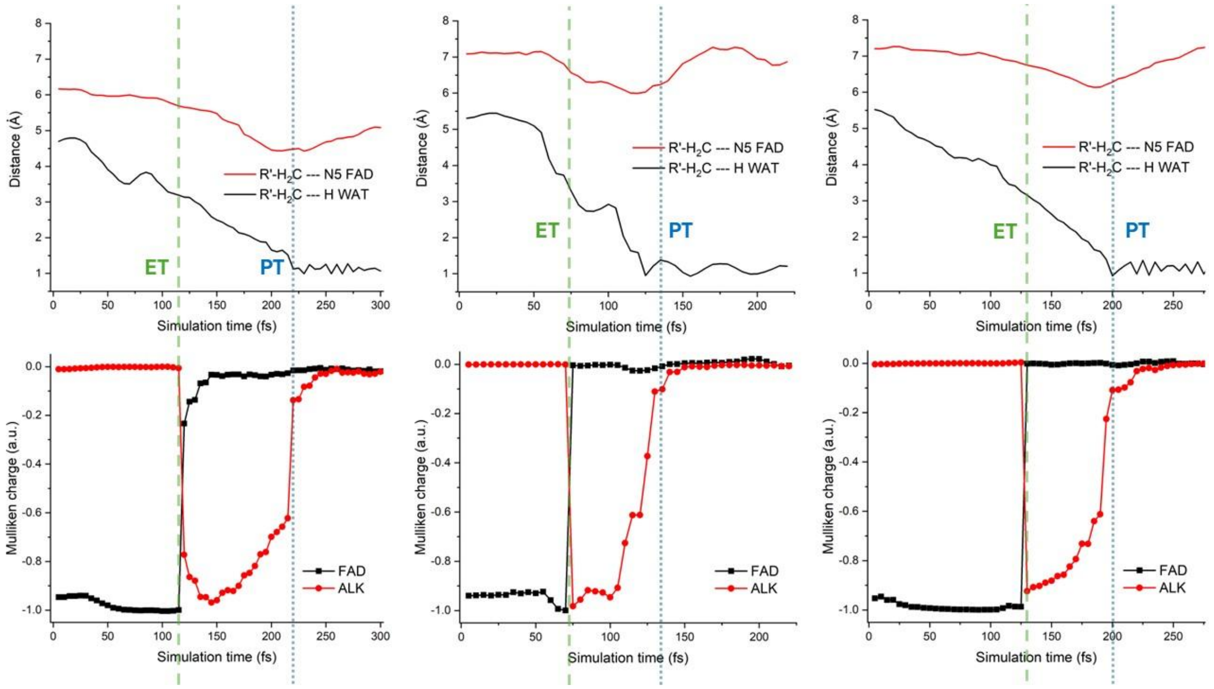

Figure S24: Analysis of three representative trajectories of scenario B, where a water molecule is involved in the PT to the alkyl chain. In the topmost panels, the distances between the sp^2 carbon atom of the alkyl chain and the water's hydrogen (in black) and the N5 atom of FAD (in red) as a function of the QM/AMOEBA MD simulation time. In the bottom panels, the Mulliken charge localized on the flavin core (in black) and the alkyl chain (in red). The back electron transfer event is highlighted by the vertical dashed green line, while the proton transfer by the vertical blue dotted line. Calculations performed at the unrestricted QM/AMOEBA level of theory by using ωB97X-D/6-31G(d).

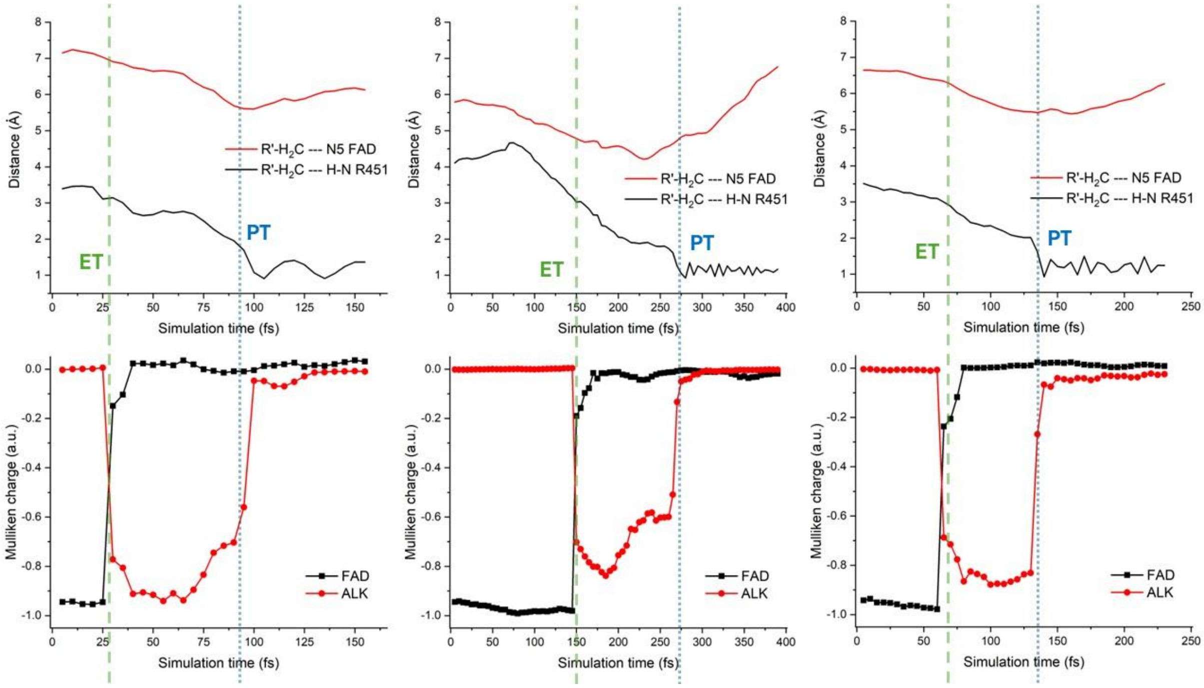

Figure S25: Analysis of three representative trajectories of scenario B, where R451 is involved in the PT to the alkyl chain. In the topmost panels, the distances between the sp^2 carbon atom of the alkyl chain and the guanidinium group's hydrogen (in black) and the N5 atom of FAD (in red) as a function of the QM/AMOEBA MD simulation time. In the bottom panels, the Mulliken charge localized on the flavin core (in black) and the alkyl chain (in red). The back electron transfer event is highlighted by the vertical dashed green line, while the proton transfer by the vertical blue dotted line. Calculations performed at the unrestricted QM/AMOEBA level of theory by using ωB97X-D/6-31G(d).

Table S1: The mean lowest excitation energy (eV) and wavelength (nm) with their relative standard deviation computed for FAD @*Cv*FAP and in scenario A and B. Level of theory: TD-DFT/AMOEBA with different functionals, basis set 6-31+G(d,p).

FAD @	Excitation energy (eV)	Excitation wavelength (nm)
	OT ωB97X-D	
***Cv*FAP**	3.10 ± 0.03	400 ± 4
**Scenario A**	3.03 ± 0.07	410 ± 10
**Scenario B**	3.07 ± 0.07	404 ± 9
	CAM-B3LYP	
***Cv*FAP**	3.17 ± 0.03	391 ± 4
**Scenario A**	3.11 ± 0.07	398 ± 9
**Scenario B**	3.15 ± 0.07	394 ± 8
	B3LYP	
***Cv*FAP**	2.84 ± 0.04	436 ± 6
**Scenario A**	2.77 ± 0.07	448 ± 11
**Scenario B**	2.81 ± 0.07	441 ± 11

References

(1) Sorigué, D.; Hadjidemetriou, K.; Blangy, S.; Gotthard, G.; Bonvalet, A.; Coquelle, N.; Samire, P.; Aleksandrov, A.; Antonucci, L.; Benachir, A.; Boutet, S.; Byrdin, M.; Cammarata, M.; Carbajo, S.; Cuiné, S.; Doak, R. B.; Foucar, L.; Gorel, A.; Grünbein, M.; Hartmann, E.; Hienerwadel, R.; Hilpert, M.; Kloos, M.; Lane, T. J.; Légeret, B.; Legrand, P.; Li-Beisson, Y.; Moulin, S. L. Y.; Nurizzo, D.; Peltier, G.; Schirò, G.; Shoeman, R. L.; Sliwa, M.; Solinas, X.; Zhuang, B.; Barends, T. R. M.; Colletier, J. P.; Joffre, M.; Royant, A.; Berthomieu, C.; Weik, M.; Domratcheva, T.; Brettel, K.; Vos, M. H.; Schlichting, I.; Arnoux, P.; Müller, P.; Beisson, F. Mechanism and Dynamics of Fatty Acid Photodecarboxylase. *Science,* **2021**, *372*, eabd5687.

(2) Georgescu, R. E.; Alexov, E. G.; Gunner, M. R. Combining Conformational Flexibility and Continuum Electrostatics for Calculating PKas in Proteins. *Biophys. J.* **2002**, *83* (4), 1731–1748.

(3) Song, Y.; Mao, J.; Gunner, M. R. MCCE2: Improving Protein PKa Calculations with Extensive Side Chain Rotamer Sampling. *J. Comput. Chem.* **2009**, *30* (14), 2231–2247.

(4) Case, D. A.; Aktulga, H. M.; Belfon, K.; Ben-Shalom, I. Y.; Berryman, J. T.; Brozell, S. R.; Cerutti, D. S.; Cheatham III, T. E.; Cisneros, G. A.; Cruzeiro, V. W. D.; Darden, T. A.; Duke, R. E.; Giambasu, G.; Gilson, M. K.; Gohlke, H.; Goetz, A. W.; Harris, R.; Izadi, A.; Izmailov, S. A.; Kasavajhala, K.; Kaymak, M. C.; King, E.; Kovalenko, A.; Kurtzman, T.; Lee, T. S.; LeGrand, S.; Li, P.; Lin, C.; Liu, J.; Luchko, T.; Luo, R.; Machado, M.; Man, V.; Manathunga, M.; Merz, K. M.; Miao, Y.; Mikhailovskii, O.; Monard, G.; Nguyen, H.; O'Hearn, K. A.; Onufriev, A.; Pan, F.; Pantano, S.; Qi, R.; Rahnamoun, A.; Roe, D. R.; Roitberg, A.; Sagui, C.; Schott-Verdugo, S.; Shajan, A.; Shen, J.; Simmerling, C. L.; Skrynnikov, N. R.; Smith, J.; Swails, J.; Walker, R. C.; Wang, J.; Wang, J.; Wei, H.; Wolf, R. M.; Wu, X.; Xiong, Y.; Xue, Y.; York, D. M.; Zhao, S.; Kollman, P. A. Amber 2022. University of California, San Francisco 2022.

(5) Tomasi, J.; Mennucci, B.; Cammi, R. Quantum Mechanical Continuum Solvation Models. *Chem. Rev.* **2005**, *105* (8), 2999–3094.

(6) Debiec, K. T.; Cerutti, D. S.; Baker, L. R.; Gronenborn, A. M.; Case, D. A.; Chong, L. T. Further along the Road Less Traveled: AMBER Ff15ipq, an Original Protein Force Field Built on a Self-Consistent Physical Model. *J. Chem. Theory Comput.* **2016**, *12* (8), 3926–3947.

(7) Berendsen, H. J. C.; Grigera, J. R.; Straatsma, T. P. The Missing Term in Effective Pair Potentials. *J. Phys. Chem.* **1987**, *91* (24), 6269–6271.

(8) Wang, J.; Wolf, R. M.; Caldwell, J. W.; Kollman, P. A.; Case, D. A. Development and Testing of a General Amber Force Field. *J. Comput. Chem.* **2004**, *25* (9), 1157–1174.

(9) Schneider, C.; Sühnel, J. A Molecular Dynamics Simulation of the Flavin Mononucleotide–RNA Aptamer Complex. *Biopolymers* **1999**, *50* (3), 287–302.

(10) Meagher, K. L.; Redman, L. T.; Carlson, H. A. Development of Polyphosphate Parameters for Use with the AMBER Force Field. *J. Comput. Chem.* **2003**, *24* (9), 1016–1025.

(11) Ponder, J. W.; Wu, C.; Ren, P.; Pande, V. S.; Chodera, J. D.; Schnieders, M. J.; Haque, I.; Mobley, D. L.; Lambrecht, D. S.; DiStasio, R. A. J.; Head-Gordon, M.; Clark, G. N. I.; Johnson, M. E.; Head-Gordon, T. Current Status of the AMOEBA Polarizable Force Field. *J. Phys. Chem. B* **2010**, *114* (8), 2549–2564.

(12) Ren, P.; Wu, C.; Ponder, J. W. Polarizable Atomic Multipole-Based Molecular Mechanics for Organic Molecules. *J. Chem. Theory Comput.* **2011**, *7* (10), 3143–3161.

(13) Bussi, G.; Donadio, D.; Parrinello, M. Canonical Sampling through Velocity Rescaling. *J. Chem. Phys.* **2007**, *126* (1), 14101.

(14) Lagardère, L.; Jolly, L.-H.; Lipparini, F.; Aviat, F.; Stamm, B.; Jing, Z. F.; Harger, M.; Torabifard, H.; Cisneros, G. A.; Schnieders, M. J.; Gresh, N.; Maday, Y.; Ren, P. Y.; Ponder, J. W.; Piquemal, J.-P. Tinker-HP: A Massively Parallel Molecular Dynamics Package for Multiscale Simulations of Large Complex Systems with Advanced Point Dipole Polarizable Force Fields. *Chem. Sci.* **2018**, *9* (4), 956–972.

(15) Frisch, M. J.; Trucks, G. W.; Schlegel, H. B.; Scuseria, G. E.; Robb, M. A.; Cheeseman, J. R.; Scalmani, G.; Barone, V.; Petersson, G. A.; Nakatsuji, H.; Li, X.; Caricato, M.; Marenich, A. V.; Bloino, J.; Janesko, B. G.; Gomperts, R.; Mennucci, B.; Hratchian, H. P.; Ortiz, J. V.; Izmaylov, A. F.; Sonnenberg, J. L.; Williams-Young, D.; Ding, F.; Lipparini, F.; Egidi, F.; Goings, J.; Peng, B.; Petrone, A.; Henderson, T.; Ranasinghe, D.; Zakrzewski, V. G.; Gao, J.; Rega, N.; Zheng, G.; Liang, W.; Hada, M.; Ehara, M.; Toyota, K.; Fukuda, R.; Hasegawa, J.; Ishida, M.; Nakajima, T.; Honda, Y.; Kitao, O.; Nakai, H.; Vreven, T.; Throssell, K.; Montgomery, J. A., J.; Peralta, J. E.; Ogliaro, F.; Bearpark, M. J.; Heyd, J. J.; Brothers, E. N.; Kudin, K. N.; Staroverov, V. N.; Keith, T. A.; Kobayashi, R.; Normand, J.; Raghavachari, K.; Rendell, A. P.; Burant, J. C.; Iyengar, S. S.; Tomasi, J.; Cossi, M.; Millam, J. M.; Klene, M.; Adamo, C.; Cammi, R.; Ochterski, J. W.; Martin, R. L.; Morokuma, K.; Farkas, O.; Foresman, J. B.; Fox, D. J. Gaussian Development Version, Revision J.19. Gaussian, Inc., Wallingford CT 2020.

(16) Pes, F.; Polack, É.; Mazzeo, P.; Dusson, G.; Stamm, B.; Lipparini, F. A Quasi Time-Reversible Scheme Based on Density Matrix Extrapolation on the Grassmann Manifold for Born–Oppenheimer Molecular Dynamics. *J. Phys. Chem. Lett.* **2023**, *14* (43), 9720–9726.

(17) Chung, L. W.; Sameera, W. M. C.; Ramozzi, R.; Page, A. J.; Hatanaka, M.; Petrova, G. P.; Harris, T. V; Li, X.; Ke, Z.; Liu, F.; Li, H.-B.; Ding, L.; Morokuma, K. The ONIOM Method and Its Applications. *Chem. Rev.* **2015**, *115* (12), 5678–5796.

(18) Frisch, M. J.; Trucks, G. W.; Schlegel, H. B.; Scuseria, G. E.; Robb, M. A.; Cheeseman, J. R.; Scalmani, G.; Barone, V.; Petersson, G. A.; Nakatsuji, H.; Li, X.; Caricato, M.; Marenich, A. V.; Bloino, J.; Janesko, B. G.; Gomperts, R.; Mennucci, B.; Hratchian, H. P.; Ortiz, J. V.; Izmaylov, A. F.; Sonnenberg, J. L.; Williams-Young, D.; Ding, F.; Lipparini, F.; Egidi, F.; Goings, J.; Peng, B.; Petrone, A.; Henderson, T.; Ranasinghe, D.; Zakrzewski, V. G.; Gao, J.; Rega, N.; Zheng, G.; Liang, W.; Hada, M.; Ehara, M.; Toyota, K.; Fukuda, R.; Hasegawa, J.; Ishida, M.; Nakajima, T.; Honda, Y.; Kitao, O.; Nakai, H.; Vreven, T.; Throssell, K.; Montgomery, J. A., J.; Peralta, J. E.; Ogliaro, F.; Bearpark, M. J.; Heyd, J. J.; Brothers, E. N.; Kudin, K. N.; Staroverov, V. N.; Keith, T. A.; Kobayashi,

R.; Normand, J.; Raghavachari, K.; Rendell, A. P.; Burant, J. C.; Iyengar, S. S.; Tomasi, J.; Cossi, M.; Millam, J. M.; Klene, M.; Adamo, C.; Cammi, R.; Ochterski, J. W.; Martin, R. L.; Morokuma, K.; Farkas, O.; Foresman, J. B.; Fox, D. J. Gaussian 16, Revision A.03. Gaussian, Inc., Wallingford CT 2016.

(19) Lipparini, F. General Linear Scaling Implementation of Polarizable Embedding Schemes. *J. Chem. Theory Comput.* **2019**, *15* (8), 4312–4317.

(20) Refaely-Abramson, S.; Sharifzadeh, S.; Jain, M.; Baer, R.; Neaton, J. B.; Kronik, L. Gap Renormalization of Molecular Crystals from Density-Functional Theory. *Phys. Rev. B - Condens. Matter Mater. Phys.* **2013**, *88* (8), 1–5.

(21) Zheng, Z.; Egger, D. A.; Brédas, J. L.; Kronik, L.; Coropceanu, V. Effect of Solid-State Polarization on Charge-Transfer Excitations and Transport Levels at Organic Interfaces from a Screened Range-Separated Hybrid Functional. *J. Phys. Chem. Lett.* **2017**, *8* (14), 3277–3283.

(22) John, C.; Pedraza-González, L.; Betti, E.; Cupellini, L.; Mennucci, B. A Computational Approach to Modeling Excitation Energy Transfer and Quenching in Light-Harvesting Complexes. *J. Phys. Chem. B* **2025**, *129* (1), 117–127.

(23) Cascone, M.; Mazzeo, P.; Cupellini, L.; Mennucci, B. Multiscale Simulation of Photoinduced Electron Transfer in Cryptochrome 4 from European Robin and Pigeon Indicates a Conserved Dynamics. *J. Phys. Chem. Lett.* **2025**, *16* (34), 8877–8884.

(24) Troisi, A.; Orlandi, G. The Hole Transfer in DNA: Calculation of Electron Coupling between Close Bases. *Chem. Phys. Lett.* **2001**, *344* (5), 509–518.

(25) Londi, G.; Salvadori, G.; Mazzeo, P.; Cupellini, L.; Mennucci, B. Protein-Driven Electron-Transfer Process in a Fatty Acid Photodecarboxylase. *JACS Au* **2025**, *5* (1), 158–168.

(26) Dell'Orletta, G.; Di Fonte, N.; Farina, M.; Daidone, I. Insights into Substrate Protonation and Solvent Accessibility in the Active Site of Fatty Acid Photodecarboxylase. *J. Phys. Chem. Lett.* **2025**, *16* (49), 12538–12544.

(27) Zhuang, B.; Liebl, U.; Vos, M. H. Flavoprotein Photochemistry: Fundamental Processes and Photocatalytic Perspectives. *J. Phys. Chem. B* **2022**, *126* (17), 3199–3207.

(28) Piano, V.; Palfey, B. A.; Mattevi, A. Flavins as Covalent Catalysts: New Mechanisms Emerge. *Trends Biochem. Sci.* **2017**, *42* (6), 457–469.

(29) He, Y.; Barone, M.; Meech, S. R.; Lukacs, A.; Tonge, P. J. Light-Driven Enzyme Catalysis: Ultrafast Mechanisms and Biochemical Implications. *Biochemistry* **2025**, *64* (12), 2491–2505.

(30) Schmermund, L.; Jurkaš, V.; Özgen, F. F.; Barone, G. D.; Büchsenschütz, H. C.; Winkler, C. K.; Schmidt, S.; Kourist, R.; Kroutil, W. Photo-Biocatalysis: Biotransformations in the Presence of Light. *ACS Catal.* **2019**, *9* (5), 4115–4144.

(31) Heyes, D. J.; Lakavath, B.; Hardman, S. J. O.; Sakuma, M.; Hedison, T. M.; Scrutton, N. S. Photochemical Mechanism of Light-Driven Fatty Acid Photodecarboxylase. *ACS Catal.*

2020, *10* (12), 6691–6696.
